# Neurogranin and VILIP-1 as Molecular Indicators of Neurodegeneration in Alzheimer’s Disease: A Systematic Review and Meta-Analysis

**DOI:** 10.3390/ijms21218335

**Published:** 2020-11-06

**Authors:** Maciej Dulewicz, Agnieszka Kulczyńska-Przybik, Barbara Mroczko

**Affiliations:** 1 Department of Neurodegeneration Diagnostics, Medical University of Bialystok, 15-269 Bialystok, Poland; agnieszka.kulczynska-przybik@umb.edu.pl (A.K.-P.); mroczko@umb.edu.pl (B.M.); 2 Department of Biochemical Diagnostics, Medical University of Bialystok, 15-269 Bialystok, Poland

**Keywords:** Alzheimer’s disease, meta-analysis, systematic review, neurogranin, visinin-like-protein-1

## Abstract

Neurogranin (Ng) and visinin-like protein 1 (VILIP-1) are promising candidates for Alzheimer’s Disease (AD) biomarkers closely related to synaptic and neuronal degeneration. Both proteins are involved in calcium-mediated pathways. The meta-analysis was performed in random effects based on the ratio of means (RoM) with calculated pooled effect size. The diagnostic utility of these proteins was examined in cerebrospinal fluid (CSF) of patients in different stages of AD compared to control (CTRL). Ng concentration was also checked in various groups with positive (+) and negative (-) amyloid beta (Aβ). Ng highest levels of RoM were observed in the AD (*n* = 1894) compared to CTRL (*n* = 2051) group (RoM: 1.62). Similarly, the VILIP-1 highest values of RoM were detected in the AD (*n* = 706) compared to CTRL (*n* = 862) group (RoM: 1.34). Concentrations of both proteins increased in more advanced stages of AD. However, Ng seems to be an earlier biomarker for the assessment of cognitive impairment. Ng appears to be related with amyloid beta, and the highest levels of Ng in CSF was observed in the group with pathological Aβ+ status. Our meta-analysis confirms that Ng and VILIP-1 can be useful CSF biomarkers in differential diagnosis and monitoring progression of cognitive decline. Although, an additional advantage of the protein concentration Ng is the possibility of using it to predict the risk of developing cognitive impairment in normal controls with pathological levels of Aβ1-42. Analyses in larger cohorts are needed, particularly concerning Aβ status.

## 1. Introduction

Alzheimer’s disease (AD) is a progressive, incurable and fatal neurodegenerative condition characterised by continuing cognitive decline. The main difficulty lies in identifying the disease in a preclinical state [1]. The onset of AD is very difficult to recognise since cognitive deficits appear much later than neuropathological changes in the brain [1,2]. Currently, Alzheimer’s disease is defined and diagnosed based on the presence of amyloid-β (Aβ) plaques and neurofibrillary tangles. Cellular and molecular changes in the brain are not yet fully understood, and classical biomarkers such as tTau, pTau181 and Aβ1-42 do not provide a full explanation of the pathogenesis of the condition. Currently, high hopes are associated with biochemical research on biomarkers which would enable earlier recognition of pathological changes. One of the most common neuropathologies in neurodegenerative disorders is disrupted synaptic transmission, which leads to the development of cognitive impairment [3]. In the initial stage of AD, called mild cognitive impairment (MCI), the most common manifestations are memory deficits [4]. Early memory deficits and other cognitive symptoms have a neuronal and molecular background closely related to synaptic plasticity, signalling or transmembrane transport and their dysfunctions [1].

The body of research on the role and importance of synaptic proteins in AD pathology increases every year. Neurogranin (Ng) and visinin-like protein 1 (VILIP-1) have been well studied as candidates for AD cerebrospinal fluid biomarkers closely related to synaptic and neuronal degeneration [5]. Ng is a post-synaptic substrate for protein kinase C (PKC). Its main function is the regulation of long-term potentiation (LTP) signalling through binding to calmodulin (CaM) [6]. Ng is mainly located in dendrites and dendritic spines in many brain structures crucial for cognitive function [7]. A number of researchers have reported that the level of Ng is increased in cerebrospinal fluid (CSF) of patients with MCI and AD compared to controls [4,8,9,10,11,12,13,14,15,16,17,18,19,20,21,22,23,24,25,26,27,28,29,30,31]. Elevated Ng levels in CSF and decreased Ng concentrations in brain tissue of patients with AD might indicate the intensity of synaptic loss and destruction [7,10,32]. Ng levels were found to be positively correlated with the concentrations of t-tau and p-tau 181 biomarkers. Although there was no clear evidence of correlations between Ng and Aβ or Mini-Mental State Examination (MMSE), differentiation between subgroups according to positive (+) or negative (-) Aβ status in AD and MCI was statistically significant. 

VILIP-1 has been identified as a biomarker of neuronal injury [33,34]. This neuronal calcium-sensor protein is widely expressed in neurons, although, similarly to Ng, its levels are reduced in the brain tissue and elevated in the CSF of patients with AD. Disturbance of Ca2+ homeostasis in neurons contributes to the neurotoxic effect of VILIP-1. Many studies have demonstrated elevated CSF concentration of VILIP-1 in patients with AD and MCI in comparison to controls [33,34,35,36,37,38,39,40,41,42,43]. VILIP-1, similarly to Ng, is strongly correlated with p-Tau 181 and t-tau. Furthermore, in contrast to Ng, it is correlated with MMSE [40], which may indicate its usefulness as a potential biomarker for monitoring cognitive decline. 

In the present meta-analysis and systematic review, we screened databases for promising synaptic and neuronal biomarkers reflecting neurodegeneration in patients in different stages of dementia due to Alzheimer’s disease. We also aimed to analyse the association between levels of Ng and VILIP-1 and disease severity, and assess the usefulness of these proteins in early diagnosis of AD. 

## 2. Results

### 2.1. Dataset Characteristics and Groups

Our literature search resulted in 315 records for Ng and 110 for VILIP-1 (Supplementary Table S1). Based on the title and abstract, 74 publications for Ng and 29 articles for VILIP-1 were selected for review. Data regarding Ng were obtained for 6517 individuals (AD (*n* = 1894), AD+ (*n* = 238), MCI (*n* = 1208), MCI+ (*n* = 430), MCI- (*n* = 241), stable MCI (sMCI) (*n* = 170), MCI due to AD (MCI-AD) (*n* = 285), control (CTRL) (*n* = 2051), CTRL+ (*n* = 103), CTRL- (*n* = 187)) and for VILIP-1 for 1761 individuals (AD (*n* = 706), MCI (*n* = 193), CTRL (*n* = 862)) from selected articles (Table 1). Subjects with lower, pathological levels of Aβ-42 and Aβ42/40 ratio below the established cut-off values ((Aβ 42 < 192 pg/mL) [13,18] and Aβ42/40 ratio < 0.063 [25]), were named as positive (Aβ+, AD+, MCI+ and CTRL+), and those with higher levels (above established cut-off values) of the mentioned biomarkers as negative, Aβ- [13,18]. 

### 2.2. Ng and VILIP-1 Measurement

Ng concentration was measured in CSF using three different quantitative methods: electrochemiluminescence (ECL) (*n* = 12), ELISA in-house (*n* = 11) and Errena Singulex (*n* = 3). The most commonly used antibody for Ng was Ng7 (epitope including amino acids 52–65) and truncated p75 (G62–P75). VILIP-1 was measured in CSF using three different quantitative methods: ELISA kits (*n* = 6), Single Molecule Counting Immunoassay (*n* = 4) and electrochemiluminescence (MSD) (*n* = 1). Values were reported in picograms per millilitre or nanograms per litre.

### 2.3. CSF Neurogranin in AD and MCI Groups

**Figure 1:**
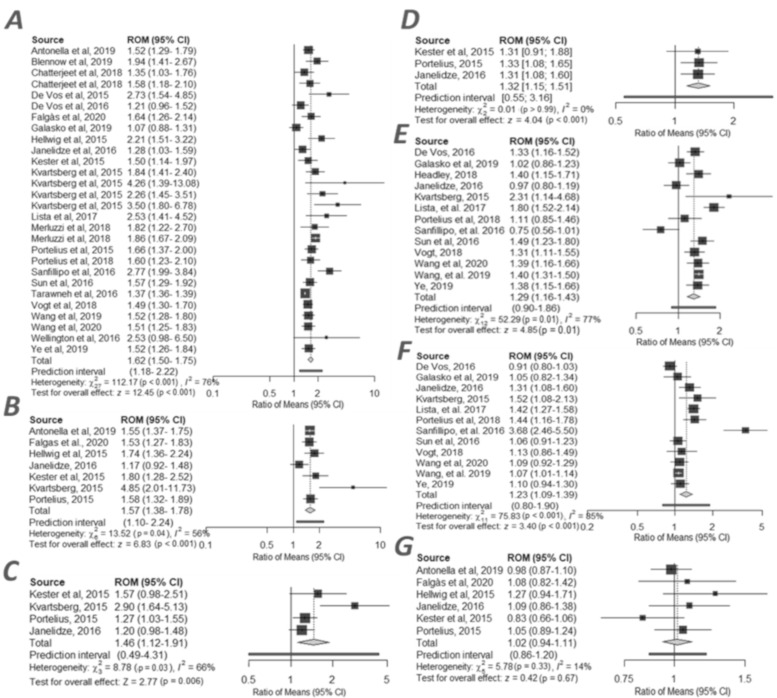
Forest plots of cerebrospinal fluid neurogranin (Ng) ratio in compared groups: (**A**) AD vs. CTRL [5,8,9,10,11,12,14,15,16,17,19,20,21,23,24,26,27,28,29,30,31,43,49,50]; (**B**) MCI-AD vs. CTRL [5,8,9,10,14,29,31]; (**C**) MCI-AD vs. sMCI [5,9,10,14]; (**D**) AD vs. sMCI [5,9,14]; (**E**) MCI vs. CTRL [4,9,10,11,15,16,17,21,24,28,30,49,50]; (**F**) AD vs. MCI [9,10,11,15,16,17,21,24,28,30,49,50]; (**G**) AD vs. MCI-AD [5,8,9,14,29,31]. Individual studies and their corresponding 95% Confidence Intervals (CIs) are indicated by filled squares. All average ratios and their corresponding 95% Cls are indicated by grey diamonds.

Ng concentrations in CSF were reported for 28 cohorts from (*n* = 24) studies. The studies included 1894 patients with AD and 2051 controls. Ng was significantly elevated in patients with AD (*n* = 1894) in comparison to controls (*n* = 2051), and the differences were largest in that group (RoM: 1.62, 95% Confidence Intervals (CI) (1.50 to 1.75), z = 12.45, *p* < 0.001) (Figure 1A) (Supplementary Figure S1, Supplementary Table S3 (1.A)). Smaller differences were observed in 7 studies with an MCI-AD group (*n* = 285) compared to CTRL (*n* = 345), with the average value of 1.57, 95% CI (1.38 to 1.78), z = 6.83, *p* < 0.001 (Figure 1B) (Supplementary Figure S2, Supplementary Table S3 (1.B)). Moderate differences were observed in 4 studies with an MCI-AD group (*n* = 285) compared to sMCI (*n* = 170), with the average value of 1.46, 95% CI (1.12–1.91, z = 2.77), *p* < 0.001 (Figure 1C) (Supplementary Figure S3, Supplementary Table S3 (1.C)), and in 3 studies with AD (*n* = 234) compared to sMCI (*n* = 147), with the average value of 1.32, 95% CI (1.15 to 1.51), z = 4.04, *p* < 0.01 (Figure 1D) (Supplementary Figure S4, Supplementary Table S3 (1.D)). Lower ratio of means was observed in 13 studies with MCI (*n* = 1280) compared to CTRL (*n* = 1167), with the average value of 1.29, 95% CI (1.11 to 1.52), z = 3.26, *p* < 0.001 (Figure 1E) (Supplementary Figure S5, Supplementary Table S3 (1.E)) and the lowest ratio of means in 12 studies with AD (*n* = 1017) compared to MCI (*n* = 1087), with the average value of 1.23, 95% CI (1.09 to 1.39), z = 3.40, *p* < 0.001 (Figure 1F) (Supplementary Figure S6, Supplementary Table S3 (1.F)). No statistically significant differences were observed between AD and MCI-AD groups (1.02, 95% CI (0.94 to 1.11), z = 0.42, *p* < 0.67) (Figure 1G) (Supplementary Figure S7, Supplementary Table S3 (1.G)). Results from all meta-analyses are presented in forest plots (Figure 1). General heterogeneity of the compared groups was high (Supplementary Table S3 (1.A–G)). All funnel plots suggested publication bias and are presented in Supplementary Figures S1–S7.

We decided to examine whether dividing the most numerous group (AD vs. CTRL) according to the type of method utilised would influence on RoM results and heterogeneity ($I^{2}$). Firstly, we divided the comparison group into two subgroups depending on the type of method used: electrochemiluminescence (*n* = 10) (ECL) and ELISA (*n* = 11). We had to exclude two studies in which Errena Singulex was used since the method was employed in only those studies [5,35]. The results demonstrated that the group of studies in which ECL was used (*n* = 11) had no heterogeneity ($I^{2}$= 25%, *p* = 0.21) and the average ratio was 1.64, 95% CI (1.53 to 1.76), z = 13.91, *p* < 0.001 (Supplementary Figures S8 and S9, and Table S3 (2)). In the group of studies in which ELISA was used (*n* = 15), higher heterogeneity ($I^{2}$ = 76%, *p* < 0.001) and impact on the result of RoM was observed (1.70, 95% CI (1.43 to 1.93), z = 6.33, *p* < 0.001 (Supplementary Figures S10 and S11, and Table S3 (3)). 

The second analysis of possible factors that may have had an impact on variation in results concerned the captured antibodies, regardless of the method employed. We selected the two most common antibodies: Ng7 (G52–G65) (*n* = 18) and Ng (G62–P75) (*n* = 3). We had to exclude three studies in which two different antibodies were used, Ng7 (G53–64) [27] and Ng (G49–G60) (P-4793) [5,35], due to too small a number of articles to enable a comparison to be made. The 4 cohorts from 3 articles in which Ng was used (G62–P75) had no heterogeneity ($I^{2}$ = 42%, *p* < 0.16) and the average level of RoM was (1.26, 95% CI (1.07 to 1.48), z = 2.83, *p* < 0.005 (Supplementary Figures S12 and S13, and Table S3 (4)). The second group of cohorts (*n* = 21), with the most commonly used type of antibody, Ng7 (G52–G65), showed $I^{2}$ heterogenity of results ($I^{2}$ = 55%, *p* < 0.001) and the highest level of RoM (1.73, 95% CI (1.59 to 1.88), z = 12.83, *p* < 0.001) (Supplementary Figures S14 and S15, and Table S3 (5)).

### 2.4. CSF Ng Levels Dependent on Aβ Status

**Figure 2:**
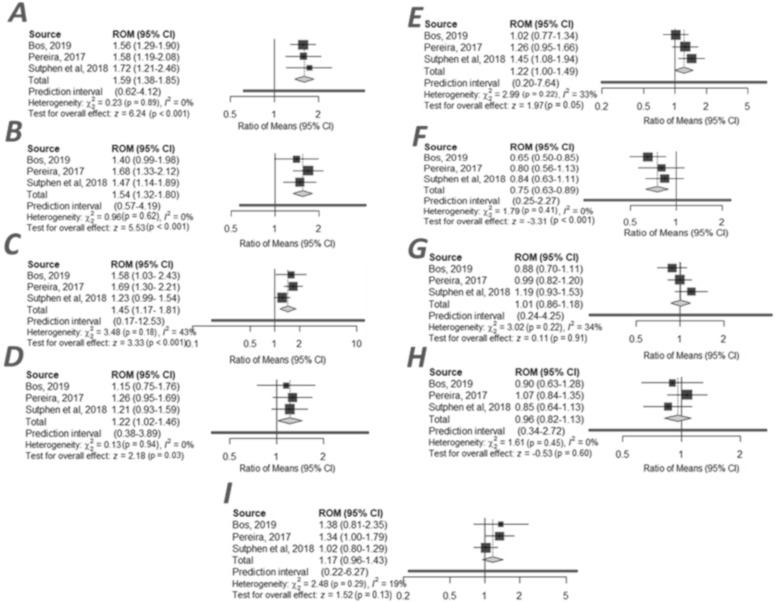
Forest plots of cerebrospinal fluid neurogranin ratio in compared groups according to amyloid beta status: (**A**) AD+ vs. MCI- [13,18,25]; (**B**) AD+ vs. CTRL- [13,18,25]; (**C**) MCI+ vs. CTRL- [13,18,25]; (**D**) MCI+ vs. CTRL+ [13,18,25]; (**E**) AD+ vs. CTRL+ [13,18,25]; (**F**) MCI- vs. CTRL+ [13,18,25]; (**G**) AD+ vs. MCI+ [13,18,25]; (**H**) MCI- vs. CTRL- [13,18,25]; (**I**) CTRL- vs. CTRL+ [13,18,25]. Individiual studies and their corresponding 95% Confidence Intervals (CIs) are indicated by filled squares. All average ratios and their corresponding 95% Cls are indicated by grey diamonds.

The smallest group of studies in the present meta-analysis included studies (*n* = 3) in which Ng concentrations were analysed in subgroups of individuals according to their positive or negative Aβ status. The greatest differences relating to elevated Ng levels in CSF were observed in the AD+ group (*n* = 238) compared to MCI- (*n* = 241) (RoM: 1.59, 95% CI (1.38 to 1.85), z = 6.24, *p* < 0.001) (Figure 2A) (Supplementary Figure S16, Supplementary Table S3 (6.A)). Marginally smaller differences in Ng levels were observed between the AD+ (*n* = 238) and CTRL- (*n* = 187) groups (1.54, 95% CI (1.32 to 1.80), z = 5.53, *p* < 0.001) (Figure 2B) (Supplementary Figure S17, Supplementary Table S3 (6.B)) as well as between patients in the MCI+ (*n* = 430) and CTRL- (*n* = 187) groups (1.45, 95% CI (1.17 to 1.81), z = 3.33, *p* < 0.001) (Figure 2C) (Supplementary Figure S18, Supplementary Table S3 (6.C)). A moderate level of RoM was observed in MCI+ (*n* = 430) compared to CTRL+ (*n* = 103) (1.22, 95% CI (1.02 to 1.46), z = 2.18, *p* < 0.03) (Figure 2D) (Supplementary Figure S19, Supplementary Table S3 (6.D)) and in AD+ (*n* = 238) compared to CTRL+ (*n* = 103) (1.22, 95% CI (1.00 to 1.49), z = 1.97, *p* < 0.05) (Figure 2E) (Supplementary Figure S20, Supplementary Table S3 (6.E)). The lowest level was observed in MCI- (*n* = 241) compared to CTRL+ (*n* = 103) 0.75, 95% CI (0.63 to 0.89), z = –3.31, *p* < 0.001 (Figure 2F) (Supplementary Figure S21, Supplementary Table S3 (6.F)). In the three compared groups, (Figure 2G) AD+ (*n* = 238) to MCI+ (*n* = 430) (1.01, 95% CI (0.86 to 1.18), z = 0.11, *p* < 0.91) (Supplementary Figure S22, Supplementary Table S3 (6.G)), (Figure 2H) MCI- (*n* = 241) to CTRL- (*n* = 187) 0.96, 95% CI (0.82 to 1.13), z = –0.53, *p* < 0.60 (Supplementary Figure S23, Supplementary Table S3 (6.H)), (Figure 2I) CTRL+ (*n* = 103) vs. CTRL- (*n* = 187), on average 1.17, 95% Cl (0.96 to 1.43), z = 1.52, *p* = 0.13 (Supplementary Figure S24, Supplementary Table S3 (6.I)), there were no statistical significant differences. Results from this meta-analysis are presented in forest plots (Figure 2). The heterogeneity of the present meta-analysis was low and with no publication bias (Supplementary Figures S16–S24) (Supplementary Table S3 (6.A–I)).

### 2.5. CSF VILIP-1 in AD and MCI Group

**Figure 3:**
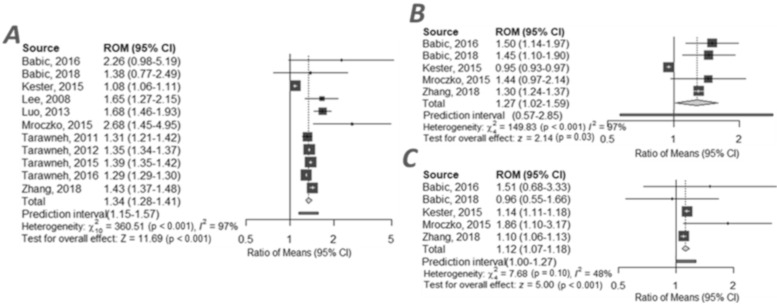
Forest plots of cerebrospinal fluid visinin-like protein 1 ratio in compared groups: (**A**) AD vs. CTRL [20,33,34,35,36,37,38,39,40,41,42]; (**B**) AD vs. MCI [33,35,40,41,42], (**C**) MCI vs. CTRL [33,35,40,41,42]. Individiual studies and their corresponding 95% Confidence Intervals (CIs) are indicated by filled squares. All average ratios and their corresponiding 95% Cls are indicated by grey diamonds.

VILIP-1 is recognised as a biomarker of neuronal degeneration. Eligible studies reporting VILIP-1 concentrations in CSF included 11 cohorts of patients with AD (*n* = 595) and CTRL (*n* = 893), and gave an average ratio of 1.34, 95% CI (1.28 to 1.41), z = 11.69, *p* < 0.001 (Figure 3A) (Supplementary Figure S25, Supplementary Table S3 (7.A)). Analysis of the AD (*n* = 336) group compared to the MCI (*n* = 193) group based on 5 cohorts revealed that the ratios were above 1 with an average of 1.27, 95% CI (1.02 to 1.59), z = 2.14, *p* < 0.03 (Figure 3B) (Supplementary Figure S26, Supplementary Table S3 (7.B)). When MCI (*n* = 193) was compared to CTRL (*n* = 105), RoM was 1.12, 95% CI (1.07 to 1.18), z = 5.00, *p* < 0.001 (Figure 3C) (Supplementary Figure S27, Supplementary Table S3 (7.C)). All results from this meta-analysis are presented in forest plots (Figure 3). In the present meta-analysis, heterogeneity was high and moderate (Supplementary Table S3 (3.A–C)).

## 3. Discussion

Our study is the most comprehensive meta-analysis of synaptic and neuronal proteins such as Ng and VILIP-1 in different stages of Alzheimer’s disease, including MCI, sMCI, MCI-AD and AD, published to date. Furthermore, we are the first researchers to perform a meta-analysis of Ng concentrations in groups of subjects depending on their amyloid-β status (Figure 2). Ng levels dependent on Aβ status may prove to be of particular importance in predicting cognitive decline in normal individuals or controls with Aβ pathology. However, we must emphasise the fact that further research is needed in CTRL+ and CTRL-. Research on these groups may allow for definitive conclusions regarding Ng as a biomarker reflecting pathological changes in preclinical stages of AD to be drawn. Literature data reveal that concentrations of Ng and VILIP-1 increase with AD severity and may therefore be useful as diagnostic biomarkers for differentiation and monitoring of disease progression [20]. However, Ng appears to be a more adequate biomarker for recognising early stages of dementia due to AD [3]. 

One of the leading causes of disturbed long-term potentiation LTP are exogenous Aβ oligomers (Aβo) which may impact on glutamate excitotoxicity or abnormalities in the calcium and calmodulin signalling pathway [55]. Two of the crucial processes related to memory, remembering and learning are long-term potentiation (LTP) and long-term depression (LTD) [24]. LTP and LTD have been extensively studied in experimental conditions and animal models as crucial factors in the development of neurodegenerative diseases, including AD [24]. The fundamental role of LTP in memory mechanisms depends on many factors, such as the Ca2+ signalling pathway, N-methyl-D-aspartate (NMDA) and α-amino-3-hydroxy-5-methyl-4-isoxazolepropionic acid (AMPA) receptors, protein kinase C (PKC), Ca2+/calmodulin-dependent protein kinase II (CaMKII) and synaptic proteins, e.g., neurogranin (Ng) [6,55]. According to the calcium hypothesis, disruption in Ca2+ signalling and synaptic dysfunction is frequently attributed to Amyloid β (Aβ) [56]. This small peptide has high propensity to aggregate in the form of senile plaques. The insoluble Aβ plaques may accumulate in the synaptic clefts, blocking LTP and inducting synaptic dysfunction, with many pathological consequences. Accumulation of Aβ oligomers appears to lead to dysfunction, loss of synaptic connections and neuronal death, which is closely related to cognitive and memory deficits [57].

Interestingly, synaptic loss is one of the earliest indicators of disease onset, which probably precedes to neuronal cell death [57]. Synaptic proteins are sought in CSF and other fluids to better understand synaptic dysfunction and its role in the pathology and progression of AD. Furthermore, innovative techniques, including mass spectrometry, liquid biopsy or super resolution microscopy, enhance the possibility of discovering novel proteins related to neuropathological processes. Literature data indicate that novel synaptic proteins such as: Calsyntetin-1 (CLSTN-1) [50], Glutamate receptor 4 (GluR4) [57], Neurexin-2A (Nrxn2a) [55,57], Neurexin-3A (Nrxn3a) [55,57], Syntaxin-1B (STX1B) [57], Thy-1 [57], Synucleins [57], Neuronal Pentraxins 1 [55], 2 [58,59] and receptor [60] (NPTX1, NPTX2, NPTXR), Synaptotagmin-1 (SYT-1) [61], Vesicle-associated membrane protein 2 (VAMP-2) [57], Synaptic vesicle glycoprotein 2A (Sv2A) [62], Contactin-2 (Cntn2) [12], Neuroligin 1 (Nlgn1) [57] and many others [63,64], related to AD and MCI, can be valuable and additional candidates for biomarkers of these diseases. Leo et al. [57] investigated changes in synaptic proteins which may precede clinical symptoms and changes in concentrations of other markers of neurodegeneration. They revealed that 6 synaptic proteins including: CLSTN-1, GluR4, NXRN2A, NRXN3A, STX1B and Thy-1, exhibit clinical usefulness in the evaluation of disease progression, particularly in periclinal stages of AD [57]. However, the authors suggested that Ng, SNAP-25 and synaptotagmin seem to be better predictors of neurodegeneration than other synaptic proteins (GluR2, Neurexin-2A, Neuroligin-2, Syntaxin-1B and VAMP-2) [57]. Considering that synapse loss and neuronal loss are interrelated in AD, it has been suggested that panels of proteins reflecting both processes should be assessed [43]. 

According to our knowledge, one of the best-studied and most promising novel synaptic proteins seems to be neurogranin. The present meta-analysis demonstrated that Ng levels were significantly higher in AD, MCI and MCI-AD compared to controls and that they related with disease severity. Elevated Ng concentrations in CSF of patients with MCI due to AD and stable MCI indicate that Ng can be useful not only in differentiation but also in monitoring disease progression. Ng is one of the post-synaptic proteins which may influence the regulation of LTP signalling through binding to calmodulin (CaM). Ng is a type of post-synaptic substrate for protein kinase C, mainly located in dendrites and spines in brain structures such as the hippocampus [65]. A decrease in Ng levels in the brain may be the cause of dysregulation of post-synaptic signalling including LTP and Ca2+ [11]. Studies have shown that Ng strengthens long-term potentiation (LTP) and is related to post-synaptic plasticity [6]. It is highly probable that Ng regulates the dynamics of CaM in dendritic spines after slowing its diffusion and increasing its availability in the synapsis [6]. Ng targets CaM within the synapse and increases the sensitivity of the synapse to the influx of Ca2+ [6]. Therefore, Ng overexpression enhances synaptic strength, increases CaMKII activation and reduces LTP induction through the NMDAR-CaMKII pathway [6,55]. Elevated Ng concentrations in CSF of patients with AD may be a mechanism of synaptic loss compensation and a means of preserving capacity of synaptic transmission, previously disturbed by Aβ. However, further research is needed to confirm this hypothesis. The majority of available publications demonstrate an inconsistent relationship between Ng concentration and Aβ, MMSE or age of patients. There exists a strong positive correlation between Ng concentration and biomarkers, such as t-tau and p-tau181 [11,25,35], Contatin-2 [26], BACE-1 [26], VILIP-1 [20]. Despite the fact that CSF Ng concentration may be a promising biomarker for AD, its evaluation in plasma has no clinical value. Currently, there is an insufficient number of reports in the literature to allow for clarification of the relevance of plasma Ng concentration in diagnosing AD or MCI [27,30]. It has been demonstrated that there are no significant differences in plasma Ng concentrations between patients with AD and healthy controls and that there is a lack of correlation between Ng content in plasma and CSF [27,57]. Additionally, studies conducted on blood plasma neuron-derived exosomes (NDEs) have reported significantly lower Ng levels in patients with AD and MCI compared to controls [66], in contrast to elevated Ng concentrations in CSF of patients with AD. A similar trend was observed in normal older people, in whom Ng levels in plasma NDEs gradually decreased over the period of 8 years but were still far lower than the concentrations in patients with AD [67]. The authors reported that lower Ng concentration can be related to its transport from plasma to CSF [67]. Recent findings demonstrate that Ng levels in plasma NDEs can be a relevant predictor of future dementia in subjects at-risk for AD several years before disease onset [67].

It has been suggested that Ng may be one of the promising prognostic factors for neurodegenerative disorders [20]. The meta-analysis of subgroups according to Aβ status demonstrated that Ng levels were higher in AD+ compared with CTRL+, CTRL- and MCI-. Ng levels were significantly lower in MCI- compared to CTRL+, which suggests that Ng is strictly related to Aβ pathology (Figure 2). There were statistically significant differences in Ng concentrations between groups of patients with AD vs. MCI-AD, AD+ vs. MCI+, MCI- vs. CTRL- and CTRL+ vs. CTRL- (Supplementary Table S3 (6.A–I)). Higher Ng levels in individuals with positive Aβ status (+), particularly in the CTRL+ group compared to CTRL-, suggest that Ng concentration combined with the result of Aβ1-42 may be useful in predicting cognitive decline in normal people and may assist in identifying at-risk individuals [13,25]. Biochemical and neuroimaging studies have demonstrated that in patients with MCI+ and CTRL+, Ng levels correlated with cortical thinning in the right precuneus and superior frontal gyri [13]. Researchers reported that cortical thickness and elevated Ng levels may indicate observable Aβ pathology in the early stages of AD [13]. Ng appears to be a sensitive biomarker of preclinical and clinical stages of the disease [5]. The division into subgroups is important for future studies and diagnostics as well as for consideration of the APOE-e4 (+/-) in patients with AD and MCI. It would be advisable for researchers to present their results with an additional analysis of subgroups according to Aβ status.

A similar trend was observed when VILIP-1 concentrations in patients with AD compared to those with MCI and controls were analysed. Furthermore, VILIP-1 level was found to be elevated in CSF and decreased in cerebral tissue of patients with AD compared to CTRL [40]. This protein plays an essential role in neuronal signalling in response to high intracellular concentration of Ca2+. VILIP-1 modulates the cascade of signals in neurons by activation of membrane-bound specific target molecules. Interestingly, VILIP-1 is assessed in the context of neuronal damage and death due to its excitotoxicity dependent on disturbed Ca2+ homeostasis [68]. It has been indicated that VILIP-1 is involved in impaired synaptic plasticity mechanisms caused by AB plaques, but the mechanism is related to axonal damage. Moreover, this protein plays an important role in indirect regulation of synaptic transmission in glutamate-dependent neurons [68]. This upregulation of VILIP-1 linked to mGluR-dependent long-term potentiation has been crucial for neuronal excitability and synaptic plasticity [68]. Our results indicate that VILIP-1 is an important biomarker of neuronal damage and can be used to differentiate Alzheimer’s disease from MCI and CTRL. Patients with mild cognitive impairment had elevated VILIP-1 levels in CSF. More studies should be conducted on patients with different stages of AD, particularly because of very high levels of heterogeneity. These variations in results may also be due to preanalytical factors, a different type of quantifying methods or later synaptic, axonal damage similar to Tau protein. The correlation between CSF VILIP-1 and MMSE scores suggests a prognostic marker for cognitive decline in early stages of AD [36]. Only one study confrimed higher level of concentration of VILIP-1 in plasma of AD patients compared to controls [35]. Further studies are needed to confirm these results, especially using different quantifining methods.

Our systematic review and meta-analysis were based on in-house and commercial assays which are prepared for research purposes only and do not undergo clinical certification. In some Ng assays, antibodies targeting different epitopes of the same molecule were used (Table 1). Although some tests were based on C-terminal antibodies (G49–G60), truncated in P75 (G62–P75), and C-terminal with an intact tip (D78), diagnostic information was very similar, with large variability of results [69]. Our study also demonstrated that despite the use of different antibodies and methods of their detection, a general trend of increasing concentrations of the tested proteins in different groups of individuals is maintained. Nevertheless, high heterogeneity of results confirms previous observations regarding the fact that differences may arise from various detection antibodies and methods used. The lowest average RoM of 1.07 in the AD vs. CTRL group was observed in one study [30]. In the study, the authors used an assay to detected C-terminal Ng truncated at P75 and reported no significant differences between AD and MCI compared to CTRL. In another study in which AD was compared with CTRL using P75, statistically significant results were obtained and had an average ratio of 1.35 for 4A and 1.58 for 4B (Table 1) [26]. These examples demonstrate that the type of antibody and method employed may have a major impact on the heterogeneity of results and differentiation. Our analysis revealed that the best results in differentiating patients with AD from CTRL were achieved by using antibodies, Ng7 (G52–G65), and the ECL method. A lack of heterogeneity of results in the meta-analysis (ECL method) (Supplementary Figures S15 and S16, and Supplementary Table S3 (2.A)) may result not only from the sensitivity of the method used but may also be due to the fact that in this group, only Ng7 (G52–G65) captured antibodies were used. Another critical factor that may have influenced the positive results of the ECL meta-analysis may be the type of plate platform reader used. For the ECL method, all researchers used the Meso Scale Discovery platform and similar procedures of development assays. By contrast, in the meta-analysis of the ELISA method, two types of antibodies: Ng7 (G52–G65) and Ng (G62–P75), were used. Another reason for the variability of results may be patient selection and the specificity of disease progression or other pre-analytical factors.

In several studies, carefully selected patients and volunteers from Alzheimer’s Disease Neuroimaging Initiative cohort (ADNI) were examined, which reduces the possibility of generalising the findings to other populations. This limitation is significant not only in relation to published results but also to the present meta-analysis. Therefore, we could not establish which particular patients were included in the study and whether they were not included in other investigations. Admittedly, in studies which used ADNI cohorts, the number of patients was never the same, but this does not exclude the possibility of repeating the results. To estimate the impact of ADNI data on the results of the present meta-analysis, we would need more detailed data on each patient from the authors of the publications. One study investigated Ng concentration in CSF of Early-Onset AD (EOAD) and demonstrated that Ng level was significantly higher in CSF of patients with AD [29]. To explain differences in Ng concentration between patients with EOAD and those with late-onset AD (LOAD), an additional analysis would be required. However, there are not sufficient data in the available literature to enable such an analysis. As for other diseases, such as Creutzfeldt-Jakob disease (CJD), higher Ng concentrations in CSF compared to AD and CTRL were reported in two studies [8,19]. The example of CJD demonstrates that Ng is a significant biomarker of synapse damage which, nonetheless, is probably not specific for AD. Expanding the existing panel of classical biomarkers by including Ng is supported not only by this meta-analysis, but also by neurophysiological and biochemical research [70,71].

In the present meta-analysis and systematic review, we aimed to summarise research results regarding two promising biomarkers—synaptic Ng and neuronal VILIP-1—which are related to neurodegeneration and pathogenesis of AD. Elevated Ng concentrations in CSF of patients with AD may be due to impaired synaptic [12] signalling that occurs earlier than changes dependent on calcium-sensor protein (VILIP-1) within the neuronal cytoplasm. Enhanced VILIP-1 levels in CSF of patients with AD and MCI compared to controls reflect progressive axonal degeneration and indicate the usefulness of VILIP-1 concentration in monitoring cognitive impairments. Importantly, Ng concentration combined with the result of amyloid status may allow for identification of individuals at a higher risk of developing neurodegenerative changes. Ng levels may allow for the stratification of patients with cognitive impairments into a group with earlier progression.

### Limitation of the Study 

Our approach is, to a certain extent, a compromise between what we were able to demonstrate and a traditional meta-analysis based on absolute concentrations and definite cut-off concentration values. Unfortunately, cut-off points for Ng and VILIP-1 have not yet been determined. However, we hope that this paper may be an important reason for their development and use of Ng as a biomarker for AD. Our meta-analysis was limited to the results of available and shared data from various authors. Restricting our search to English language publications may have excluded some relevant studies. Small groups of patients with the MCI and Aβ status may have also had a negative impact on the effect size. Strong heterogeneity of results only indicates a general trend of protein concentration elevation in different stages of the disease. However, this general trend was not confirmed in one study [30]. Due to a lack of access to raw data on MMSE and age of patients, additional meta-regression or linear mixed models could not be performed. Several researchers have reported diagnostic utility of Ng in predicting future cognitive impairment in healthy individuals and cognitive decline in AD [20].

## 4. Materials and Methods 

### 4.1. Search Strategy

**Figure 4:**
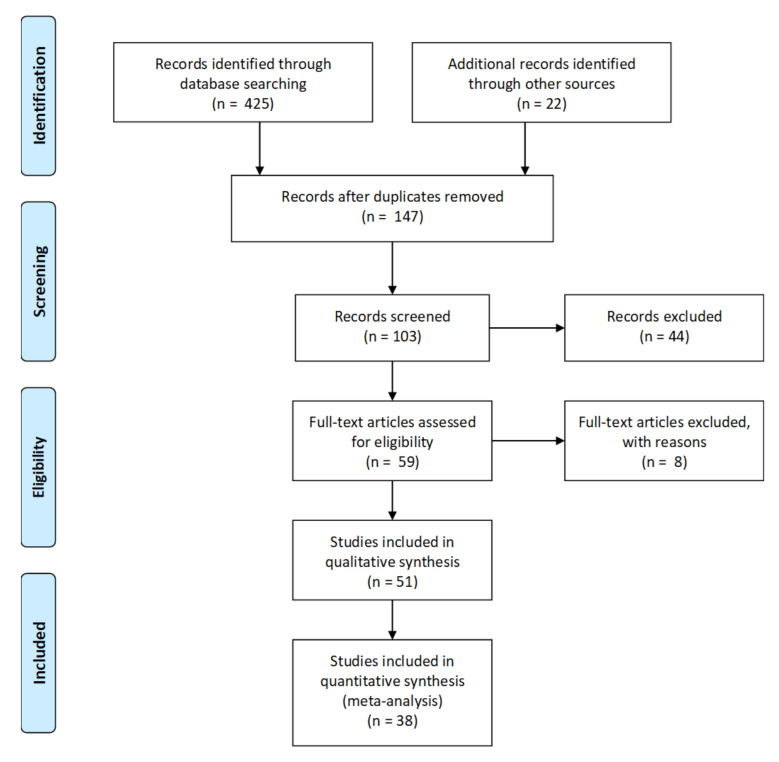
Flow diagram of the study-selection process used for the meta-analysis of Ng and VILIP-1.

This systematic review and meta-analysis were performed in accordance with the Preferred Reporting Items for Systematic Reviews and Meta-Analysis (PRISMA) reporting guidelines (Figure 4). The databases: Scopus, Web of Science and PubMed, were searched (using search terms ‘Neurogranin’ AND ‘Alzheimer’s Disease’, ‘VILIP-1’ AND ‘Alzheimer’s Disease’) for original articles published in the English language between January 1990 and 20 March 2020 (Supplementary Table S1). Other websites with conference abstracts, databases, e.g., Cochrane Library, were searched using these phrases. The quality of articles was assessed using relevant criteria from the Quality of Diagnostic Accuracy Research Studies (QUADAS) guidelines. In all materials, information regarding study approval by the local ethics committee was checked. All abstracts were reviewed and selected against relevant inclusion criteria (Supplementary Table S2).

### 4.2. Inclusion Criteria

Original articles were included if lumbar cerebrospinal fluid (CSF) levels of Ng and/or VILIP-1 were analysed by quantifying methods in neurological patients with Alzheimer’s disease (AD), mild cognitive impairments (MCI), stable mild cognitive impairments (sMCI), mild cognitive impairments due to Alzheimer’s disease (MCI-AD) and controls (CTRL). The study group consisted of patients clearly defined on diagnostic criteria (The International Working Group (IWG-2) criteria [51], Albert et al., 2011 [45], McKhann et al., 2011 [44], Petersen et al., 2004 [47], Petersen et al., 1999 [48], McKhann et al., 1984 [46], Dubois et al., 2007 [52], Morris et al., 2006 [53], Berg et al., 1998 [54]). The number of subjects was established at >10 in the experimental group and >8 individuals in the CTRL group. Additionally, we checked the Mini-Mental State Exam score (MMSE). Only articles where the following ranges of the MMSE score were used for groups were included: AD between 18 to 23, MCI between 23 to 27 and CTRL higher than 27. If the diagnostic criteria or the MMSE scores were not reported, relevant information regarding patients was checked and entered in the Supplementary Table S2. 

To date, no reference or cut-off values have been established for Ng and VILIP-1 since these proteins are still considered potentially novel candidates for AD biomarkers. CSF concentration of these proteins is measured using quantifying methods of human CSF such as ELISA kit, In-house ELISA, xMAP, Electrochemiluminescence (ECL), Microparticle-based immunoassay (MBI) Singulex Erenna, Single molecule array (Simoa™) and others. The mean values and standard deviation (SD) were not combined in the analysed studies, even when the authors (one article) presented two or more cohorts or subgroups according to the Aβ status (Aβ+ or Aβ-). All relevant distinctions are marked (*) in Table 1.

We excluded review, opinion and other articles in which the reported levels of Ng and VILIP-1 did not have necessary data, including the control group or values, presented only in graphical form. We excluded articles with experimental animal and computational models. 

### 4.3. Data Collection

Data on mean and SD, age, diagnosis and MMSE scores were extracted from the publications or requested from the corresponding author. In the majority of papers regarding biomarkers, authors present median values with 25th and 75th quartiles. This type of data does not allow for the performance of a meta-analysis. For three articles, we used a quantile method for estimating X and S based on Scenario C3 [72]. Only after converting to an estimated mean and SD were other tests and forest plots performed. The articles with calculated estimated means are marked by (*) next to the number (Table 1). Finally 28 studies were selected and included in the present meta-analysis (*n* = 28) [4,5,8,9,11,12,13,15,16,17,18,19,20,21,24,25,26,28,29,30,31,43,49,50], with data from six of them (*n* = 6) [5,10,14,23,27,43] obtained from Alzforum (https://www.alzforum.org/alzbiomarker). As for VILIP-1, 11 studies (*n* = 11) [20,33,34,35,36,37,38,39,40,41,42] were selected and included in this meta-analysis. Results from data extraction included: quality assessment questions (QUADAS 1–13), The PubMed Identifier (PMID) numbers, name of journal, first author, type of methods, type of control groups, additional important information, type of antibodies and diagnostic criteria, which are reported in Supplementary Table S2. 

All information was collected in order to account for what may have affected the large variety of results in published articles. Calculation of mean differences is not sufficient to disregard the problems of variability (e.g., different cut-points for biomarker concentrations, various protocols and methods or different antibodies) which we addressed in the discussion section. To reduce these problems, we did meta-analysis using ratio of mean (RoM) concentration biomarkers. 

### 4.4. Statistical Analysis

All calculations and visualisation of data were performed using R Studio (v. 1.2.5033) with package ‘meta’, ‘metaphor’. Both proteins were rated by random-effect meta-analysis based on ratio of means between all types of cohorts. An estimate of heterogeneity was taken from the inverse-variance random-effect model by DerSimonian and Laird [73]. We calculated effect size based on the weighted average of each study. A test for overall effect was performed (z-score). Therefore, the effect size (ES) and its (95%) confidence interval (CI) allow to observe changes in the RoM. The weights of each study were determined by the method of inverse-variance and were reflected in the size of each square and lines. The RoM was selected for the present meta-analysis for several reasons, including high variation of results depending on the measurement method, different laboratories and their cut-points, different assays and antibodies. RoM of biomarkers may reduce these problems, indicating the ratio of differences between means [74]. 

## 5. Conclusions

This comprehensive meta-analysis and systematic review confirmed that higher CSF levels of Ng and VILIP-1 are associated with AD. Moreover, the concentrations of these proteins increase with disease stage (from lower in MCI through moderate in sMCI and MCI-AD to highest in patients with AD). Therefore, determination of Ng and VILIP-1 levels could be useful not only in diagnosing AD but also for monitoring disease progression. Furthermore, using Ng concentration in combination with the results of amyloid-β1-42 may create the possibility of predicting a higher risk for cognitive impairment in healthy individuals or identifying patients at an increased risk for disease progression. The use of these two proteins in combination with classic biomarkers such as tTau, pTau and Ab1-42 may increase the diagnostic sensitivity of tests. 

## Figures and Tables

**Table 1:** Datasets included in the meta-analysis.

Neurogranin (Ng)							
N.	Source	Diagnostic Categories	Controls (CTRL)	Diagnostic Criteria	Method	Type of Capture Antibody	PMID
1	Antonell et al., 2019 [8]	AD (*n* = 102); MCI-AD (*n* = 56)	(*n* = 47)	McKhann et al., 2011 [44]; Albert et al., 2011 [45]	ELISA In-house	Ng7 (G52–G65)	31668967
2	Blennow et al., 2019 [19]	AD (*n* = 46)	(*n* = 64)	McKhann et al., 1984 [46]	ECL In-house (MSD)	Ng7 (G52–65)	31097472
3	Bos et al., 2019 [25]	AD+ (*n* = 157); MCI+ (*n* = 263); MCI- (*n* = 187)	Aβ+ (*n* = 45); Aβ- (*n* = 95)	McKhann et al., 1984 [46]; Petersen, 2004 [47]	ECL In-house (MSD)	Ng7 (G52–G65)	30853464
4A	Chatterjeet et al., 2018 [26]	AD (*n* = 70)	(*n* = 20)	McKhann et al., 2011 [44]	ELISA kit Euroimmun	Ng (G62-P75)	29859129
4B	Chatterjeet et al., 2018 [26]	AD (*n* = 36)	(*n* = 28)	McKhann et al., 2011 [44]	ELISA kit Euroimmun	Ng (G62-P75)	29859129
5	De Vos et al., 2015 [27]	AD (*n* = 20)	(*n* = 29)	McKhann et al., 2011 [44]	ELISA In-house	Ng7 (G53–64)	26092348
6	De Vos et al., 2016 [28]	AD (*n* = 50); MCI (*n* = 38)	(*n* = 20)	McKhann et al., 2011 [44]	ELISA In-house	Ng (G62-P75)	27392859
7	Falgàs et al., 2020 [29]	AD (*n* = 23); MCI-AD (*n* = 26)	(*n* = 37)	McKhann et al., 2011 [44]; Albert et al., 2011 [45]	ELISA In-house	Ng7 (G52–G65)	31944489
8	Galasko et al., 2019 [30]	AD (*n* = 46); MCI (*n* = 57)	(*n* = 90)	McKhann et al., 2011 [44]; Albert et al., 2011 [45]	ELISA kit Euroimmun	Ng (G62-P75)	31853477
9	Headley et al., 2018 [4]	MCI (*n* = 193)	(*n* = 111)	McKhann et al., 1984 [46]	ECL In-house (MSD)	Ng7 (G53–G64)	29429972
10	Hellwig et al., 2015 [31]	AD (*n* = 39); MCI-AD (*n* = 13)	(*n* = 21)	McKhann et al., 2011 [44]	ECL In-house (MSD)	Ng7 (G52–G65)	26698298
11	Janelidze et al., 2016 [9]	AD (*n* = 74); MCI-AD (*n* = 35); sMCI (*n* = 62)	(*n* = 53)	McKhann et al., 1984 [46]; Petersen, 2004 [47]	ELISA In-house	Ng7 (G52–G65)	26783546
12	Kester et al., 2015 [5]	AD (*n* = 65); MCI-AD (*n* = 36); sMCI (*n* = 17)	(*n* = 37)	McKhann et al., 1984 [46]; Petersen et al., 1999 [48]	Erenna^®^ Singulex	Ng G49-G60(P-4793)	26366630
13A	Kvartsberg et al., 2015 [10]	AD (*n* = 16)	(*n* = 10)	McKhann et al., 1984 [46]	ELISA In-house	Ng7 (G52–G65)	25533203
13B	Kvartsberg et al., 2015 [10]	AD (*n* = 44)	(*n* = 30)	McKhann et al., 1984 [46]	ELISA In-house	Ng7 (G52–G65)	25533203
13C	Kvartsberg et al., 2015 [10]	AD (*n* = 40); MCI (*n* = 40)	(*n* = 40)	McKhann et al., 1984 [46]	ELISA In-house	Ng7 (G52–G65)	25533203
13D	Kvartsberg et al., 2015 [10]	sMCI (*n* = 23); MCI-AD (*n* = 14)	(*n* = 0)	McKhann et al., 1984 [46] Petersen et al., 1999 [48] Petersen, 2004 [47]	ELISA In-house	Ng7 (G52–G65)	25533203
14	Kvartsberg et al., 2015 [43]	AD (*n* = 25)	(*n* = 20)	McKhann et al., 1984 [46]	ECL In-house (MSD)	Ng7 (G52–G65)	26136856
15 *	Lista et al., 2017 [11]	AD (*n* = 35); MCI (*n* = 41)	(*n* = 21)	McKhann et al., 2011 [44]; Albert et al., 2011 [45]	ELISA In-house	Ng7 (G52–G65)	28731449
16A	Merluzzi et al., 2018 [12]	AD (*n* = 40)	(*n* = 25)	McKhann et al., 2011 [44]	ECL In-house (MSD)	Ng7 (G52–G65)	29959263
16B	Merluzzi et al., 2018 [12]	AD (*n* = 61)	(*n* = 291)	McKhann et al., 2011 [44]	ECL In-house (MSD)	Ng7 (G52–G65)	29959263
17	Pereira et al., 2017 [13]	AD+ (*n* = 65); MCI+ (*n* = 109); MCI- (*n* = 36)	Aβ+ (*n* = 37); Aβ- (*n* = 57)	McKhann et al., 1984 [46]; Petersen, 2004 [47]	ECL In-house (MSD)	Ng7 (G52–G65)	28692877
18	Portelius et al., 2015 [14]	AD (*n* = 95); MCI-AD (*n* = 105); sMCI (*n* = 68)	(*n* = 110)	McKhann et al., 1984 [46]; Petersen, 2004 [47]	ECL In-house (MSD)	Ng7 (G52–G65)	26373605
19 *	Portelius et al., 2018 [15]	AD (*n* = 397); MCI (*n* = 114)	(*n* = 75)	McKhann et al., 2011 [44]; McKhann et al., 1984 [46]	ELISA In-house	Ng22 (epitope 63–75)	29700597
20 *	Sanfillipo et al., 2016 [16]	AD (*n* = 25); MCI (*n* = 50)	(*n* = 44)	McKhann et al., 2011 [44]	ELISA In-house	Ng7 (G52–G65)	27531278
21	Sun et al., 2016 [17]	AD (*n* = 95); MCI (*n* = 193)	(*n* = 111)	McKhann et al., 1984 [46]	ECL In-house (MSD)	Ng7 (G52–G65)	27321472
22	Sutphen et al., 2018 [18]	AD+ (*n* = 16); MCI+ (*n* = 58); MCI- (*n* = 18)	Aβ+ (*n* = 21); Aβ- (*n* = 35)	McKhann et al., 1984 [46];	Erenna^®^ Singulex	Ng G49-G60(P-4793)	29580670
23	Tarawneh et al., 2016 [20]	AD (*n* = 95)	(*n* = 207)	McKhann et al., 1984 [46]	Erenna^®^ Singulex	Ng G49-G60(P-4793)	27018940
24	Vogt et al., 2018 [21]	AD (*n* = 40); MCI (*n* = 35)	(*n* = 335)	McKhann et al., 1984 [46]; Albert et al., 2011 [45]	ECL In-house (MSD)	Ng7 (G52–G65)	30579367
25	Wang et al., 2020 [49]	AD (*n* = 67); MCI (*n* = 143)	(*n* = 47)	McKhann et al., 1984 [46]	ECL In-house (MSD)	Ng7 (G52–G65)	32021212
26 *	Wang, et al., 2019 [50]	AD (*n* = 81); MCI (*n* = 171)	(*n* = 99)	McKhann et al., 1984 [46]	ECL In-house (MSD)	Ng7 (G52–G65)	29667155
27	Wellington et al., 2016 [23]	AD (*n* = 100)	(*n* = 19)	McKhann et al., 1984 [46]	ELISA In-house	Ng7 (G52–G65)	26826204
28	Ye et al., 2019 [24]	AD (*n* = 67); MCI (*n* = 143)	(*n* = 84)	IWG-2 [51]	ECL In-house (MSD)	Ng7 (G52–G65)	30447377
**Visinin-like protein 1 (VILIP-1)**							
1.	Babic et al., 2016 [41]	AD (*n* = 109); MCI (*n* = 43)	(*n* = 9)	McKhann et al., 1984 [46] Petersen et al., 1999 [48]	ELISA kit		26836160
2.	Babic et al., 2018 [35]	AD (*n* = 111); MCI (*n* = 50)	(*n* = 9)	McKhann et al., 1984 [46] Petersen et al., 1999 [48] Albert et al., 2011 [45]	ELISA kit		30329219
3.	Kester et al., 2015 [33]	AD (*n* = 65); MCI (*n* = 61)	(*n* = 37)	McKhann et al., 1984 [46]	ELISA kit		26383836
4.	Lee et al., 2008 [34]	AD (*n* = 33)	(*n* = 24)	McKhann et al., 1984 [46]	ECL In-house (MSD)		18703769
5.	Luo et al., 2013 [38]	AD (*n* = 61)	(*n* = 40)	Dubois et al., 2007 [52]	ELISA kit		23800322
6.	Mroczko et al., 2015 [40]	AD (*n* = 33); MCI (*n* = 15)	(*n* = 18)	McKhann et al., 2011 [44]	ELISA kit		25159667
7.	Tarawneh et al., 2011 [36]	AD (*n* = 98)	(*n* = 211)	Morris et al., 2006 [53]; Berg et al., 1998 [54]	MBI Erenna^®^ Singulex		21823155
8.	Tarawneh et al., 2012 [37]	AD (*n* = 60)	(*n* = 211)	Morris et al., 2006 [53]; Berg et al., 1998 [54]	MBI Erenna^®^ Singulex		22357717
9.	Tarawneh et al., 2015 [39]	AD (*n* = 23)	(*n* = 64)	Morris et al., 2006 [53]; Berg et al., 1998 [54]	MBI Erenna^®^ Singulex		25867677
10.	Tarawneh et al., 2016 [20]	AD (*n* = 95)	(*n* = 207)	Albert et al., 2011 [45]	MBI Erenna^®^ Singulex		27018940
11.	Zhang et al., 2018 [42]	AD (*n* = 18); MCI (*n* = 24)	(*n* = 32)	McKhann et al., 1984 [46]	ELISA kit		30311914

Note—Numbers and capital letter indicate different groups or cohorts in the same article (1A cohort one and 1B cohort two). Numbers with * are studies in which the estimated average was used. The diagnostic category was entered following what the authors declared in their articles or data sent to us. More detailed information on the characteristics of the control group is presented in the Supplementary Table S2. The PubMed Identifier (PMID) is a unique number for each article. ECL—electrochemiluminescence method, MBI—Microparticle-based immunoassay for Erenna Singulex system, AD—Alzheimer’s Disease, MCI—Mild Cognitive Impairments, MCI-AD—MCI due to AD, sMCI—stable MCI.

## Supplementary Materials

Supplementary materials can be found at https://www.mdpi.com/1422-0067/21/21/8335/s1.

## References

[1] Blennow K., Zetterberg H. (2018). Biomarkers for Alzheimer’s Disease: Current Status and Prospects for the Future.

[2] Hampel H., Toschi N., Baldacci F., Zetterberg H., Blennow K., Kilimann I., Teipel S.J., Cavedo E., Melo Dos Santos A., Epelbaum S. (2018). Alzheimer’s disease biomarker-guided diagnostic workflow using the added value of six combined cerebrospinal fluid candidates: Aβ 1–42, total-tau, phosphorylated-tau, NFL, neurogranin, and YKL-40. Alzheimer’s Dement..

[3] Höglund K., Kern S., Zettergren A., Börjesson-Hansson A., Zetterberg H., Skoog I., Blennow K. (2017). Preclinical amyloid pathology biomarker positivity: Effects on tau pathology and neurodegeneration. Transl. Psychiatry.

[4] Headley A., De Leon-Benedetti A., Dong C., Levin B., Loewenstein D., Camargo C., Rundek T., Zetterberg H., Blennow K., Wright C.B. (2018). Neurogranin as a predictor of memory and executive function decline in MCI patients. Neurology.

[5] Kester M.I., Teunissen C.E., Crimmins D.L., Herries E.M., Ladenson J.H.K.H., Scheltens P., van der Flier W.M., Morris J.C., Holtzman D.M., Fagan A.M. (2015). Neurogranin as a cerebrospinal fluid biomarker for synaptic loss in symptomatic Alzheimer disease. JAMA Neurol..

[6] Petersen A., Gerges N.Z. (2015). Neurogranin regulates CaM dynamics at dendritic spines. Sci. Rep..

[7] De Wilde M.C., Overk C.R., Sijben J.W., Masliah E. (2016). Meta-analysis of synaptic pathology in Alzheimer’s disease reveals selective molecular vesicular machinery vulnerability. Alzheimer’s Dement..

[8] Antonell A., Tort-Merino A., Ríos J., Balasa M., Borrego-Écija S., Auge J.M., Muñoz-García C., Bosch B., Falgàs N., Rami L. (2020). Synaptic, axonal damage and inflammatory cerebrospinal fluid biomarkers in neurodegenerative dementias. Alzheimer’s Dement..

[9] Janelidze S., Hertze J., Zetterberg H., Landqvist Waldö M., Santillo A., Blennow K., Hansson O. (2016). Cerebrospinal fluid neurogranin and YKL-40 as biomarkers of Alzheimer’s disease. Ann. Clin. Transl. Neurol..

[10] Kvartsberg H., Duits F.H., Ingelsson M., Andreasen N., Öhrfelt A., Andersson K., Brinkmalm G., Lannfelt L., Minthon L., Hansson O. (2015). Cerebrospinal fluid levels of the synaptic protein neurogranin correlates with cognitive decline in prodromal Alzheimer’s disease. Alzheimer’s Dement..

[11] Lista S., Toschi N., Baldacci F., Zetterberg H., Blennow K., Kilimann I., Teipel S.J., Cavedo E., Dos Santos A.M., Epelbaum S. (2017). Cerebrospinal Fluid Neurogranin as a Biomarker of Neurodegenerative Diseases: A Cross-Sectional Study. J. Alzheimer’s Dis..

[12] Merluzzi A.P., Carlsson C.M., Johnson S.C., Schindler S.E., Asthana S., Blennow K., Zetterberg H., Bendlin B.B. (2018). Neurodegeneration, synaptic dysfunction, and gliosis are phenotypic of Alzheimer dementia. Neurology.

[13] Pereira J.B., Westman E., Hansson O. (2017). Association between cerebrospinal fluid and plasma neurodegeneration biomarkers with brain atrophy in Alzheimer’s disease. Neurobiol. Aging.

[14] Portelius E., Zetterberg H., Skillbäck T., Törnqvist U., Andreasson U., Trojanowski J.Q., Weiner M.W., Shaw L.M., Mattsson N., Blennow K. (2015). Cerebrospinal fluid neurogranin: Relation to cognition and neurodegeneration in Alzheimer’s disease. Brain.

[15] Portelius E., Olsson B., Höglund K., Cullen N.C., Kvartsberg H., Andreasson U., Zetterberg H., Sandelius Å., Shaw L.M., Lee V.M.Y. (2018). Cerebrospinal fluid neurogranin concentration in neurodegeneration: Relation to clinical phenotypes and neuropathology. Acta Neuropathol..

[16] Sanfilippo C., Forlenza O., Zetterberg H., Blennow K. (2016). Increased neurogranin concentrations in cerebrospinal fluid of Alzheimer’s disease and in mild cognitive impairment due to AD. J. Neural Transm..

[17] Sun X., Dong C., Levin B., Crocco E., Loewenstein D., Zetterberg H., Blennow K., Wright C.B. (2016). APOE ε4 carriers may undergo synaptic damage conferring risk of Alzheimer’s disease. Alzheimer’s Dement..

[18] Sutphen C.L., McCue L., Herries E.M., Xiong C., Ladenson J.H., Holtzman D.M., Fagan A.M. (2018). ADNI Longitudinal decreases in multiple cerebrospinal fluid biomarkers of neuronal injury in symptomatic late onset Alzheimer’s disease. Alzheimer’s Dement..

[19] Blennow K., Diaz-Lucena D., Zetterberg H., Villar-Pique A., Karch A., Vidal E., Hermann P., Schmitz M., Ferrer Abizanda I., Zerr I. (2019). CSF neurogranin as a neuronal damage marker in CJD: A comparative study with AD. J. Neurol. Neurosurg. Psychiatry.

[20] Tarawneh R., D’Angelo G., Crimmins D., Herries E., Griest T., Fagan A.M., Zipfel G.J., Ladenson J.H., Morris J.C., Holtzman D.M. (2016). Diagnostic and prognostic utility of the synaptic marker neurogranin in alzheimer disease. JAMA Neurol..

[21] Vogt N.M., Romano K.A., Darst B.F., Engelman C.D., Johnson S.C., Carlsson C.M., Asthana S., Blennow K., Zetterberg H., Bendlin B.B. (2018). The gut microbiota-derived metabolite trimethylamine N-oxide is elevated in Alzheimer’s disease. Alzheimers. Res. Ther..

[22] Nichols E., Szoeke C.E.I., Vollset S.E., Abbasi N., Abd-Allah F., Abdela J., Aichour M.T.E., Akinyemi R.O., Alahdab F., Asgedom S.W. (2018). Global, regional, and national burden of Alzheimer’s disease and other dementias, 1990–2016: A systematic analysis for the Global Burden of Disease Study 2016. Lancet Neurol..

[23] Wellington H., Paterson R.W., Portelius E., Törnqvist U., Magdalinou N., Fox N.C., Blennow K., Schott J.M., Zetterberg H. (2016). Increased CSF neurogranin concentration is specific to Alzheimer disease. Neurology.

[24] Ye X., Zhou W., Zhang J., Alzheimer’s Disease Neuroimaging Initiative (2019). Association of CSF CD40 levels and synaptic degeneration across the Alzheimer’s disease spectrum. Neurosci. Lett..

[25] Bos I., Vos S., Verhey F., Scheltens P., Teunissen C., Engelborghs S., Sleegers K., Frisoni G., Blin O., Richardson J.C. (2019). Cerebrospinal fluid biomarkers of neurodegeneration, synaptic integrity, and astroglial activation across the clinical Alzheimer’s disease spectrum. Alzheimer’s Dement..

[26] Chatterjee M., Del Campo M., Morrema T.H.J.J., de Waal M., van der Flier W.M., Hoozemans J.J.M.M., Teunissen C.E. (2018). Contactin-2, a synaptic and axonal protein, is reduced in cerebrospinal fluid and brain tissue in Alzheimer’s disease. Alzheimer’s Res. Ther..

[27] De Vos A., Jacobs D., Struyfs H., Fransen E., Andersson K., Portelius E., Andreasson U., De Surgeloose D., Hernalsteen D., Sleegers K. (2015). C-terminal neurogranin is increased in cerebrospinal fluid but unchanged in plasma in Alzheimer’s disease. Alzheimer’s Dement..

[28] De Vos A., Struyfs H., Jacobs D., Fransen E., Klewansky T., De Roeck E., Robberecht C., Van Broeckhoven C., Duyckaerts C., Engelborghs S. (2016). The cerebrospinal fluid neurogranin/BACE1 ratio is a potential correlate of cognitive decline in Alzheimer’s Disease. J. Alzheimer’s Dis..

[29] Falgàs N., Ruiz-Peris M., Pérez-Millan A., Sala-Llonch R., Antonell A., Balasa M., Borrego-Écija S., Ramos-Campoy O., Augé J.M., Castellví M. (2020). Contribution of CSF biomarkers to early-onset Alzheimer’s disease and frontotemporal dementia neuroimaging signatures. Hum. Brain Mapp..

[30] Galasko D., Xiao M., Xu D., Smirnov D., Salmon D.P., Dewit N., Vanbrabant J., Jacobs D., Vanderstichele H., Vanmechelen E. (2019). Synaptic biomarkers in CSF aid in diagnosis, correlate with cognition and predict progression in MCI and Alzheimer’s disease. Alzheimer’s Dement..

[31] Hellwig K., Kvartsberg H., Portelius E., Andreasson U., Oberstein T.J., Lewczuk P., Blennow K., Kornhuber J., Maler J.M., Zetterberg H. (2015). Neurogranin and YKL-40: Independent markers of synaptic degeneration and neuroinflammation in Alzheimer’s disease. Alzheimer’s Res. Ther..

[32] Kvartsberg H., Lashley T., Murray C.E., Brinkmalm G., Cullen N.C., Höglund K., Zetterberg H., Blennow K., Portelius E. (2018). The intact postsynaptic protein neurogranin is reduced in brain tissue from patients with familial and sporadic Alzheimer’s disease. Acta Neuropathol..

[33] Kester M.I., Teunissen C.E., Sutphen C., Herries E.M., Ladenson J.H., Xiong C., Scheltens P., van der Flier W.M., Morris J.C., Holtzman D.M. (2015). Cerebrospinal fluid VILIP-1 and YKL-40, candidate biomarkers to diagnose, predict and monitor Alzheimer’s disease in a memory clinic cohort. Alzheimers Res. Ther..

[34] Lee J.-M., Blennow K., Andreasen N., Laterza O., Modur V., Olander J., Gao F., Ohlendorf M., Ladenson J.H. (2008). The brain injury biomarker VLP-1 is increased in the cerebrospinal fluid of Alzheimer disease patients. Clin. Chem..

[35] Babić Leko M., Willumsen N., Nikolac Perković M., Klepac N., Borovečki F., Hof P.R., Sonicki Z., Pivac N., de Silva R., Šimić G. (2018). Association of MAPT haplotype-tagging polymorphisms with cerebrospinal fluid biomarkers of Alzheimer’s disease: A preliminary study in a Croatian cohort. Brain Behav..

[36] Tarawneh R., D’Angelo G., Macy E., Xiong C., Carter D., Cairns N.J., Fagan A.M., Head D., Mintun M.A., Ladenson J.H. (2011). Visinin-like protein-1: Diagnostic and prognostic biomarker in Alzheimer disease. Ann. Neurol..

[37] Tarawneh R., Lee J.-M., Ladenson J.H., Morris J.C., Holtzman D.M. (2012). CSF VILIP-1 predicts rates of cognitive decline in early Alzheimer disease. Neurology.

[38] Luo X., Hou L., Shi H., Zhong X., Zhang Y., Zheng D., Tan Y., Hu G., Mu N., Chan J. (2013). CSF levels of the neuronal injury biomarker visinin-like protein-1 in Alzheimer’s disease and dementia with Lewy bodies. J. Neurochem..

[39] Tarawneh R., Head D., Allison S., Buckles V., Fagan A.M., Ladenson J.H., Morris J.C., Holtzman D.M. (2015). Cerebrospinal Fluid markers of neurodegeneration and rates of brain atrophy in early Alzheimer Disease. JAMA Neurol..

[40] Mroczko B., Groblewska M., Zboch M., Muszyński P., Zajkowska A., Borawska R., Szmitkowski M., Kornhuber J., Lewczuk P. (2015). Evaluation of visinin-like protein 1 concentrations in the cerebrospinal fluid of patients with mild cognitive impairment as a dynamic biomarker of Alzheimer’s disease. J. Alzheimers. Dis..

[41] Babić Leko M., Borovečki F., Dejanović N., Hof P.R., Šimić G. (2016). Predictive value of cerebrospinal fluid visinin-like protein-1 levels for Alzheimer’s Disease early detection and differential diagnosis in patients with mild cognitive impairment. J. Alzheimer’s Dis..

[42] Zhang H., Ng K.P., Therriault J., Kang M.S., Pascoal T.A., Rosa-Neto P., Gauthier S. (2018). Cerebrospinal fluid phosphorylated tau, visinin-like protein-1, and chitinase-3-like protein 1 in mild cognitive impairment and Alzheimer’s disease. Transl. Neurodegener..

[43] Kvartsberg H., Portelius E., Andreasson U., Brinkmalm G., Hellwig K., Lelental N., Kornhuber J., Hansson O., Minthon L., Spitzer P. (2015). Characterization of the postsynaptic protein neurogranin in paired cerebrospinal fluid and plasma samples from Alzheimer’s disease patients and healthy controls. Alzheimer’s Res. Ther..

[44] McKhann G.M., Knopman D.S., Chertkow H., Hyman B.T., Jack C.R., Kawas C.H., Klunk W.E., Koroshetz W.J., Manly J.J., Mayeux R. (2011). The diagnosis of dementia due to Alzheimer’s disease: Recommendations from the national institute on aging-Alzheimer’s Association workgroups on diagnostic guidelines for Alzheimer’s disease. Alzheimer’s Dement..

[45] Albert M.S., DeKosky S.T., Dickson D., Dubois B., Feldman H.H., Fox N.C., Gamst A., Holtzman D.M., Jagust W.J., Petersen R.C. (2011). The diagnosis of mild cognitive impairment due to Alzheimer’s disease: Recommendations from the National Institute on Aging-Alzheimer’s Association workgroups on diagnostic guidelines for Alzheimer’s disease. Alzheimer’s Dement..

[46] McKhann G., Drachman D., Folstein M., Katzman R., Price D., Stadlan E.M. (1984). Clinical diagnosis of Alzheimer’s disease: Report of the NINCDS-ADRDA Work Group* under the auspices of Department of Health and Human Services Task Force on Alzheimer’s Disease. Neurology.

[47] Petersen R.C. (2004). Mild cognitive impairment as a diagnostic entity. J. Intern. Med..

[48] Petersen R.C., Smith G.E., Waring S.C., Ivnik R.J., Tangalos E.G., Kokmen E. (1999). Mild cognitive impairment: Clinical characterization and outcome. Arch. Neurol..

[49] Wang J., Zhang X., Zhu B., Fu P. (2020). Association of clusterin levels in cerebrospinal fluid with synaptic degeneration across the Alzheimer’s Disease continuum. Neuropsychiatr. Dis. Treat..

[50] Wang L. (2019). Association of cerebrospinal fluid Neurogranin with Alzheimer’s disease. Aging Clin. Exp. Res..

[51] Dubois B., Feldman H.H., Jacova C., Hampel H., Molinuevo J.L., Blennow K., DeKosky S.T., Gauthier S., Selkoe D., Bateman R. (2014). Advancing research diagnostic criteria for Alzheimer’s disease: The IWG-2 criteria. Lancet Neurol..

[52] Dubois B., Feldman H.H., Jacova C., DeKosky S.T., Barberger-Gateau P., Cummings J., Delacourte A., Galasko D., Gauthier S., Jicha G. (2007). Research criteria for the diagnosis of Alzheimer’s disease: Revising the NINCDS–ADRDA criteria. Lancet Neurol..

[53] Morris M.C., Evans D.A., Tangney C.C., Bienias J.L., Wilson R.S. (2006). Associations of vegetable and fruit consumption with age-related cognitive change. Neurology.

[54] Berg L., McKeel D.W., Miller J.P., Storandt M., Rubin E.H., Morris J.C., Baty J., Coats M., Norton J., Goate A.M. (1998). Clinicopathologic Studies in Cognitively Healthy Aging and Alzheimer Disease. Arch. Neurol..

[55] Zhong L., Gerges N.Z. (2012). Neurogranin targets calmodulin and lowers the threshold for the induction of long-term potentiation. PLoS ONE.

[56] Khachaturian Z.S. (2017). Calcium Hypothesis of Alzheimer’s disease and brain aging: A framework for integrating new evidence into a comprehensive theory of pathogenesis. Alzheimer’s Dement..

[57] Lleó A., Núñez-Llaves R., Alcolea D., Chiva C., Balateu-Paños D., Colom-Cadena M., Gomez-Giro G., Muñoz L., Querol-Vilaseca M., Pegueroles J. (2019). Changes in synaptic proteins precede neurodegeneration markers in preclinical Alzheimer’s Disease cerebrospinal fluid. Mol. Cell. Proteom..

[58] Vagnoni A., Perkinton M.S., Gray E.H., Francis P.T., Noble W., Miller C.C.J. (2012). Calsyntenin-1 mediates axonal transport of the amyloid precursor protein and regulates A production. Hum. Mol. Genet..

[59] Brinkmalm G., Sjödin S., Simonsen A.H., Hasselbalch S.G., Zetterberg H., Brinkmalm A., Blennow K. (2018). A Parallel reaction monitoring mass spectrometric method for analysis of potential CSF biomarkers for Alzheimer’s Disease. Proteom. Clin. Appl..

[60] Oeckl P., Metzger F., Nagl M., von Arnim C.A.F., Halbgebauer S., Steinacker P., Ludolph A.C., Otto M. (2016). Alpha-, Beta-, and Gamma-synuclein quantification in cerebrospinal fluid by multiple reaction monitoring reveals increased concentrations in Alzheimer′s and Creutzfeldt-Jakob Disease but no alteration in synucleinopathies. Mol. Cell. Proteom..

[61] Spellman D.S., Wildsmith K.R., Honigberg L.A., Tuefferd M., Baker D., Raghavan N., Nairn A.C., Croteau P., Schirm M., Allard R. (2015). Development and evaluation of a multiplexed mass spectrometry based assay for measuring candidate peptide biomarkers in Alzheimer’s Disease Neuroimaging Initiative (ADNI) CSF. Proteom. Clin. Appl..

[62] Begcevic I., Tsolaki M., Brinc D., Brown M., Martinez-Morillo E., Lazarou I., Kozori M., Tagaraki F., Nenopoulou S., Gkioka M. (2018). Neuronal pentraxin receptor-1 is a new cerebrospinal fluid biomarker of Alzheimer’s disease progression. F1000Research.

[63] Clarke M.T.M., Brinkmalm A., Foiani M.S., Woollacott I.O.C., Heller C., Heslegrave A., Keshavan A., Fox N.C., Schott J.M., Warren J.D. (2019). CSF synaptic protein concentrations are raised in those with atypical Alzheimer’s disease but not frontotemporal dementia. Alzheimer’s Res. Ther..

[64] Mecca A.P., Chen M., O’Dell R.S., Naganawa M., Toyonaga T., Godek T.A., Harris J.E., Bartlett H.H., Zhao W., Nabulsi N.B. (2020). In vivo measurement of widespread synaptic loss in Alzheimer’s disease with SV2A PET. Alzheimer’s Dement..

[65] Davidsson P., Puchades M., Blennow K. (1999). Identification of synaptic vesicle, pre- and postsynaptic proteins in human cerebrospinal fluid using liquid-phase isoelectric focusing. Electrophoresis.

[66] Goetzl E.J., Kapogiannis D., Schwartz J.B., Lobach I.V., Goetzl L., Abner E.L., Jicha G.A., Karydas A.M., Boxer A., Miller B.L. (2016). Decreased synaptic proteins in neuronal exosomes of frontotemporal dementia and Alzheimer’s disease. FASEB J..

[67] He M.F., Sun L., Cao W., Yin C., Sun W., Liu P., Tan L., Xu Z., Zhao W. (2020). Association between plasma exosome neurogranin and brain structure in patients with Alzheimer’s disease: A protocol study. BMJ Open.

[68] Braunewell K.H. (2012). The visinin-like proteins VILIP-1 and VILIP-3 in Alzheimer’s disease—Old wine in new bottles. Front. Mol. Neurosci..

[69] Willemse E.A.J.J., De Vos A., Herries E.M., Andreasson U., Engelborghs S., van der Flier W.M., Scheltens P., Crimmins D., Ladenson J.H., Vanmechelen E. (2018). Neurogranin as cerebrospinal fluid biomarker for Alzheimer disease: An assay comparison study. Clin. Chem..

[70] Li L., Lai M., Cole S., Le Novère N., Edelstein S.J. (2020). Neurogranin stimulates Ca2+/calmodulin-dependent kinase II by suppressing calcineurin activity at specific calcium spike frequencies. PLoS Comput. Biol..

[71] Zhong L., Gerges N.Z. (2020). Neurogranin Regulates Metaplasticity. Front. Mol. Neurosci..

[72] Wan X., Wang W., Liu J., Tong T. (2014). Estimating the sample mean and standard deviation from the sample size, median, range and/or interquartile range. BMC Med. Res. Methodol..

[73] DerSimonian R., Laird N. (1986). Meta-analysis in clinical trials. Control. Clin. Trials.

[74] Friedrich J.O., Adhikari N.K.J., Beyene J. (2008). The ratio of means method as an alternative to mean differences for analyzing continuous outcome variables in meta-analysis: A simulation study. BMC Med. Res. Methodol..

