# Supplementary material for: Neurogranin and VILIP-1 as Molecular Indicators of Neurodegeneration in Alzheimer’s Disease: A Systematic Review and Meta-Analysis
Source: Int J Mol Sci. 2020 Nov 6;21(21):8335. doi: 10.3390/ijms21218335 (PMC7664397; doi:10.3390/ijms21218335)

Supplementary contents for the article:

Neurogranin and VILIP-1 as indicators of neurodegeneration in Alzheimer's disease.

A systematic review and meta-analysis.

Table S1 Searching terms in databases in results with number of articles

	"Neurogranin" AND "Alzheimer's disease"	"VILIP-1" AND "Alzheimer's disease"
SCOPUS Review	34	3
SCOPUS Article	93	35
SCOPUS ALL	141	40
Web of Science Review	10	8
Web of Science Article	76	29
Web of Science ALL	88	38
PubMed Review	12	3
PubMed Article	74	29
PubMed ALL	86	32

Table S2 Supplemental content with results from data extraction.

			Neurogranin (Ng)						
	PMID	Journal	First author/Year	AD group (n)	MCI group (n)	MCI due to AD group (n)	sMCI group (n)	Controls (CTRL)	Type of CTRL group
1	31668967	Alzheimers Dement.	Antonella et al, 2019	102	x	56	x	47	Healthy controls
2	31097472	J Neurol Neurosurg Psychiatry.	Blennow et al, 2019	46	x	x	x	64	neurological controls
3	30853464	Alzheimers Dement.	Bos et al, 2019	157	AB+(n=263)/AB-(n=187)	x	x	AB+(n=45) AB- (n=95)	Healthy controls
4A	29859129	Alzheimers Res Ther.	Chatterjeet et al, 2018	36	x	x	x	28	selected from the Amsterdam Dementia Cohort)
4B	29859129	Alzheimers Res Ther.	Chatterjeet et al, 2018	70	x	x	x	20	selected from the Amsterdam Dementia Cohort)
5	26092348	Alzheimers Dement.	De Vos et al, 2015	20	x	x	x	29	controls
6	27392859	J Alzheimers Dis	De Vos et al, 2016	50	38	x	x	20	age-matched cognitively healthy elderly
7	31944489	Hum Brain Mapp.	Falgàs et al, 2020	23	x	26	x	37	Healthy controls
8	31853477	Alzheimers Dement.	Galasko et al, 2019	46	57	x	x	90	cognitively normal controls
9	29429972	Neurology.	Headley et al, 2018	x	193	x	x	111	participants with normal cognition (ADNI)
10	26698298	Alzheimers Res Ther.	Hellwig et al, 2015	39	x	13	x	21	non demented young controls (this group was not included in the original publication)
11	26783546	Ann Clin Transl Neurol.	Janelidze et al, 2016	74	x	35	62	53	cognitively healthy controls
12	26366630	JAMA Neurol.	Kester et al, 2015	65	x	36	17	37	cognitively normal participants
13A	25533203	Alzheimers Dement.	Kvartsberg et al, 2015	16	x	x	x	10	controls
13B	25533203	Alzheimers Dement.	Kvartsberg et al, 2015	44	x	x	x	30	controls
13C	25533203	Alzheimers Dement.	Kvartsberg et al, 2015	40	40	x	x	40	controls
13D	25533203	Alzheimers Dement.	Kvartsberg et al, 2015	x	x	14	23	0	controls
14	26136856	Alzheimers Res Ther.	Kvartsberg et al, 2015	25	x	x	x	20	healthy controls
15*	28731449	J Alzheimers Dis	Lista et al, 2017	35	41	x	x	21	cognitively healthy controls
16A	29959263	Neurology.	Merluzzi et al, 2018	40	x	x	x	25	unimpaired cognition were classified as controls
16B	29959263	Neurology.	Merluzzi et al, 2018	61	x	x	x	291	unimpaired cognition were classified as controls
17	28692877	Neurobiol Aging.	Pereira et al, 2017	65	AB+(n=109)/AB-(n=36)	x	x	AB+(n=37) AB- (n=57)	Healthy controls
18	26373605	Brain.	Portelius et al, 2015	95	x	105	68	110	cognitively normal subjects
19*	29700597	Acta Neuropathol.	Portelius et al, 2018	397	114	x	x	75	Controls
20*	27531278	J Neural Transm (Vienna).	Sanfillipo et al, 2016	25	50	x	x	44	cognitively normal controls
21	27321472	Alzheimers Dement.	Sun et al, 2016	95	193	x	x	111	subjects with normal cognition
22	29580670	Alzheimers Dement.	Sutphen et al, 2018	16	AB+ (n=58)/AB-(n=18)	x	x	AB+(n=21) AB- (n=35)	Healthy controls
23	27018940	JAMA Neurol.	Tarawneh et al, 2016	95	x	x	x	207	cognitively normal controls
24	30579367	Alzheimers Res Ther.	Vogt et al, 2018	40	35	x	x	335	cognitively-unimpaired individuals
25	32021212	Neuropsychiatr Dis Treat.	Wang et al, 2020	67	143	x	x	47	individuals with normal cognition
26*	29667155	Aging Clin Exp Res.	Wang, et al, 2019	81	171	x	x	99	cognitively normal
27	26826204	Neurology.	Wellington et al, 2016	100	x	x	x	19	healthy controls
28	30447377	Neurosci Lett.	Ye et al, 2019	67	143	x	x	84	normal cognition
			Visinin like protein 1 (VILIP-1)						
1.	26836160	J Alzheimers Dis.	Babic et al, 2016	109	43	x	x	9	Healthy controls
2.	30329219	Brain Behav.	Babic, 2018	111	50	x	x	9	Healthy controls
3.	26383836	Alzheimers Res Ther.	Kester et al, 2015	65	61	x	x	37	Cognitively normal
4.	18703769	Clin Chem.	Lee et al, 2008	33	x	x	x	24	Cognitively normal controls
5.	23800322	J Neurochem.	Luo et al, 2013	61	x	x	x	40	Healthy elder controls
6.	25159667	J Alzheimers Dis.	Mroczko et al, 2015	33	15	x	x	18	Elderly individuals without cognitive deficits
7.	21823155	Ann Neurol.	Tarawneh et al, 2011	98	x	x	x	211	Cognitively normal controls
8.	22357717	Neurology.	Tarawneh et al, 2012	60	x	x	x	211	Cognitively normal controls
9.	25867677	JAMA Neurol.	Tarawneh et al, 2015	23	x	x	x	64	Cognitively normal controls
10.	27018940	JAMA Neurol.	Tarawneh et al, 2016	95	x	x	x	207	Cognitively normal controls
11.	30311914	Transl Neurodegener.	Zhang et al, 2018	18	24	x	x	32	Cognitively normal from ADNI database

Table S3 Results of meta-analysis in each compared groups

Group		No. Figure	No. of studies	No. of subjects		ROM	95%CI		z-score	p	Heterogeneity			
											Q	p	H	I2
1. Meta-analysis results of CSF neurogranin levels in patients with compared groups														
A	AD vs CTRL	1 | e-1	28	1894	2051	1,62	1,5	1,75	12,15	0,001	112,17	0,001	2,04	76%
B	MCI-AD vs CTRL	1 | e-2	7	285	345	1,57	1,38	1,78	6,83	0,01	13,52	0,04	1,5	56%
C	MCI-AD vs sMCI	1 | e-3	4	285	170	1,46	1,12	1,91	2,77	0,006	8,78	0,03	1,71	66%
D	AD vs sMCI	1 | e-4	3	234	147	1,32	1,15	1,51	4,04	0,001	0,01	0,99	1	0%
E	MCI vs CTRL	1 | e-5	13	1280	1167	1,29	1,16	1,43	4,83	0,001	52,29	0,001	2,09	77%
F	AD vs MCI	1 | e-6	12	1017	1087	1,23	1,09	1,39	3,4	0,001	75,83	0,001	2,63	85%
G	AD vs MCI-AD	1 | e-7	6	398	271	1,02	0,94	1,11	0,42	0,67	5,78	0,33	1,08	14%
2. Results of meta-analysis of cerebrospinal fluid neurogranin levels using electrochemiluminescence (ECL) in patients with AD and CTRL														
AD vs CTRL		e-8-9	11	710	1199	1,64	1,53	1,76	13,91	0,001	13,32	0,21	1,15	25%
3. Results of meta-analysis of cerebrospinal fluid neurogranin levels using ELISA in patients with AD and CTRL														
AD vs CTRL		e-10-11	15	1024	608	1,7	1,46	1,99	6,72	0,001	53,92	0,001	1,96	74%
4. Results of meta-analysis of cerebrospinal fluid neurogranin levels using detection antibodies (G62-P75) in patients with AD and CTRL														
AD vs CTRL		e-12-13	4	202	158	1,26	1,07	1,48	2,83	0,005	5,17	0,16	1,31	42%
5. Results of meta-analysis of cerebrospinal fluid neurogranin levels using detection antibodies (G52-G65) in patients with AD and CTRL														
AD vs CTRL		e-14-15	21	1135	1574	1,73	1,59	1,88	12,86	0,001	44,76	0,001	1,5	55%
6. Meta-analysis results of CSF neurogranin levels in patients with compared groups dependent of Aβ status														
A	AD+ vs MCI-	2 | e-16	3	238	241	1,59	1,38	1,85	6,24	0,001	0,23	0,89	1	0%
B	AD+ vs CTRL-	2 | e-17	3	238	187	1,54	1,32	1,8	5,53	0,001	0,96	0,62	1	0%
C	MCI+ vs CTRL-	2 | e-18	3	430	187	1,45	1,17	1,81	3,33	0,001	3,48	0,18	1,32	43%
D	MCI+ vs CTRL+	2 | e-19	3	430	103	1,22	1,02	1,46	2,18	0,03	0,13	0,94	1	0%
E	AD+ vs CTRL+	2 | e-20	3	238	103	1,22	1	1,49	1,97	0,05	2,99	0,22	1,22	33%
F	MCI- vs CTRL+	2 | e-21	3	241	103	0,75	0,63	0,89	-3,31	0,001	1,79	0,41	1	0%
G	AD+ vs MCI+	2 | e-22	3	238	430	1,01	0,86	1,18	0,11	0,91	3,02	0,22	1,23	34%
H	MCI- vs CTRL-	2 | e-23	3	241	187	0,96	0,82	1,13	-0,53	0,6	1,61	0,45	1	0%
I	CTRL+ vs CTRL-	2 | e-24	3	103	187	1,17	0,96	1,43	1,52	0,13	2,48	0,29	1,11	19%
7. Meta-analysis results of CSF Visinin like protein 1 levels in patients with compared groups														
A	AD vs CTRL	3 | e-25	11	706	862	1,34	1,28	1,41	11,69	0,001	360,51	0,001	6	97%
B	AD vs MCI	3 | e-26	5	336	193	1,27	1,02	1,59	2,14	0,001	149,83	0,001	6,12	97%
C	MCI vs CTRL	3 | e-27	5	193	105	1,12	1,07	1,18	5	0,001	7,68	0,1	1,39	48%

1. Funnel plots of CSF ratios of neurogranin for each compared groups.

A

Figure S1 Funnel plot of CSF Ng in Alzheimer's disease samples vs controls

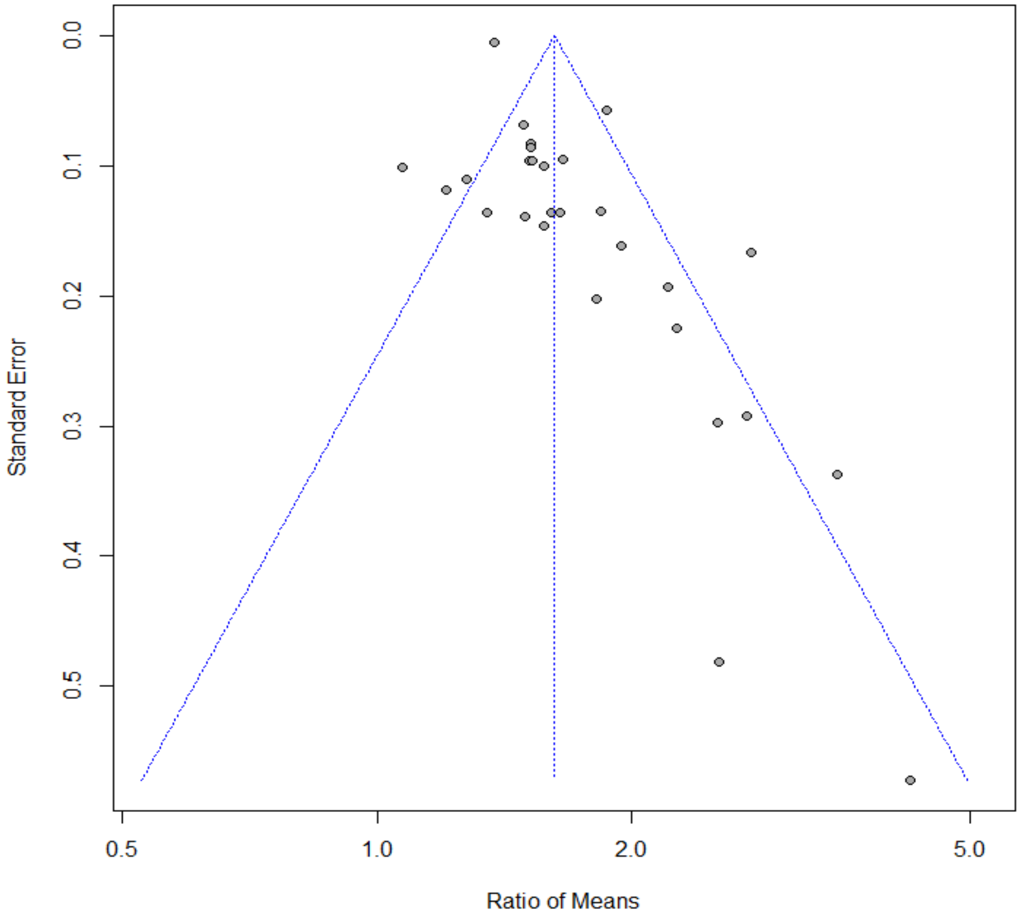

Figure S2 Funnel plot of CSF ratios of Ng between mild cognitive impairments due to Alzheimer's disease (MCI-AD) and controls (CTRL)

B

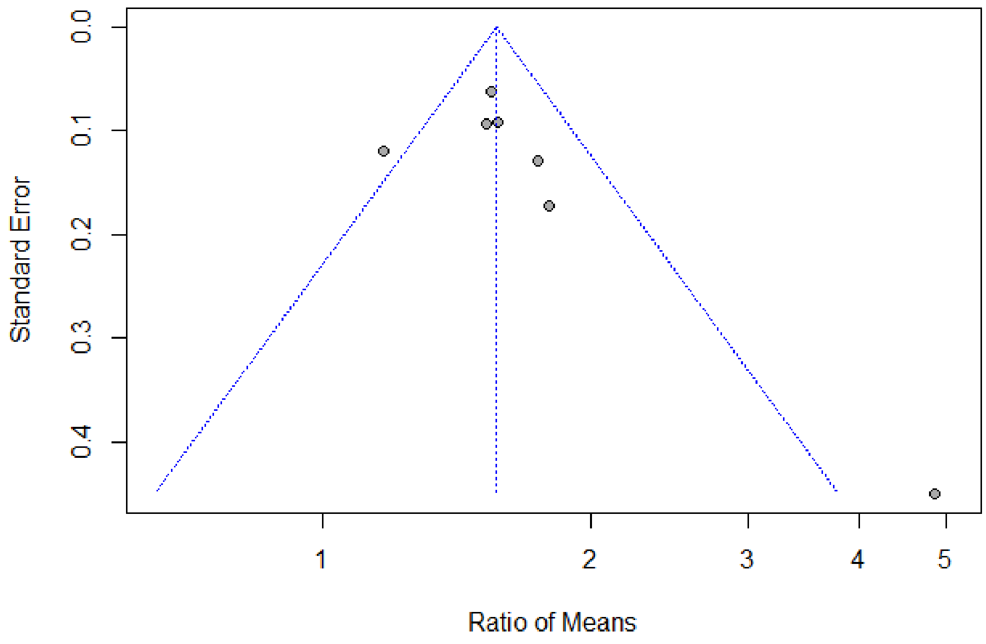

C

Figure S3 Funnel plot of CSF ratios of Ng between mild cognitive impairments due to Alzheimer's disease (MCI-AD) vs stable mild cognitive impairments (sMCI)

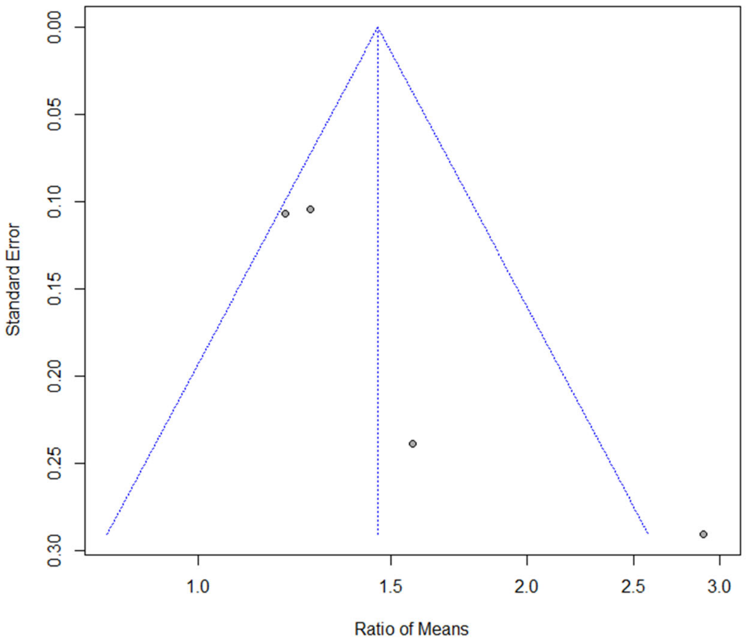

D

Figure S4 Funnel plot of CSF ratios of Ng between Alzheimer's disease (AD) and stable mild cognitive impairments (sMCI)

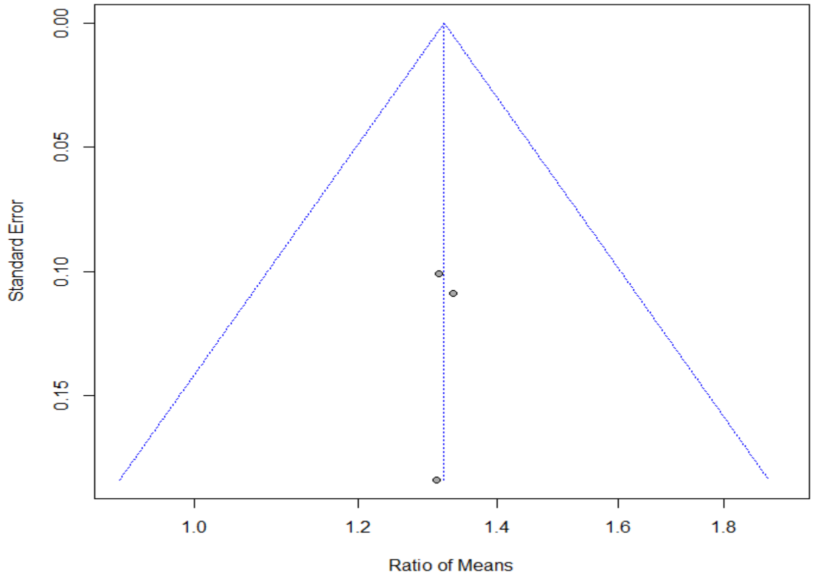

E

Figure S5 Funnel plot of CSF CSF ratios of Ng between mild cognitive impairments (MCI) vs controls (CTRL)

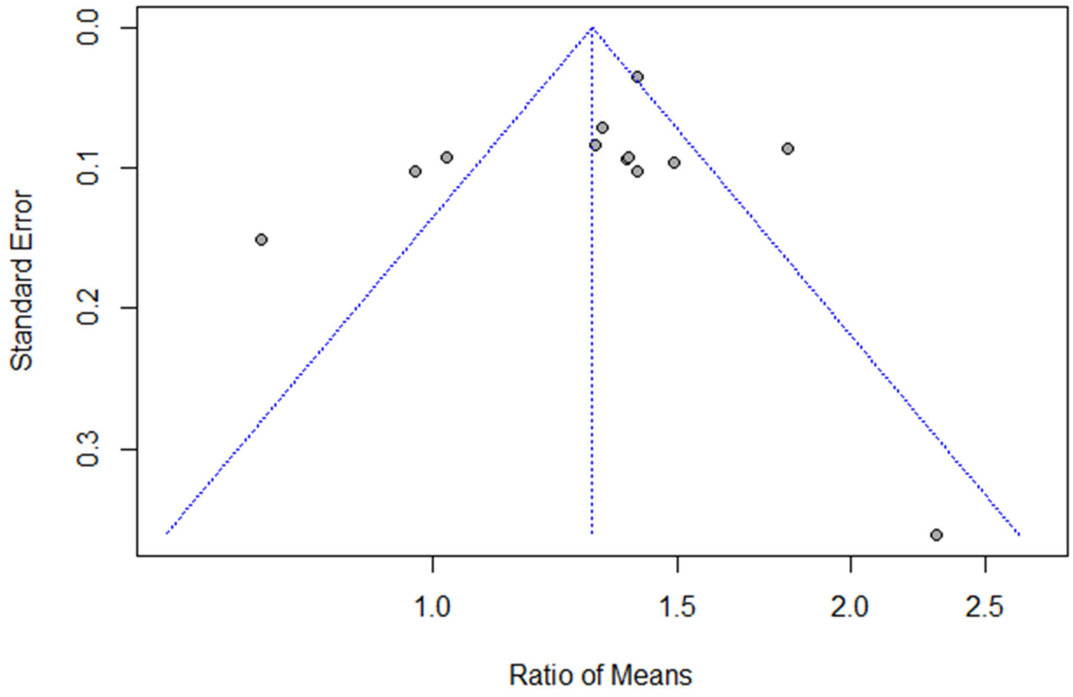

F

Figure S6 Funnel plot of CSF ratios of Ng between Alzheimer's disease (AD) vs mild cognitive impairments (MCI)

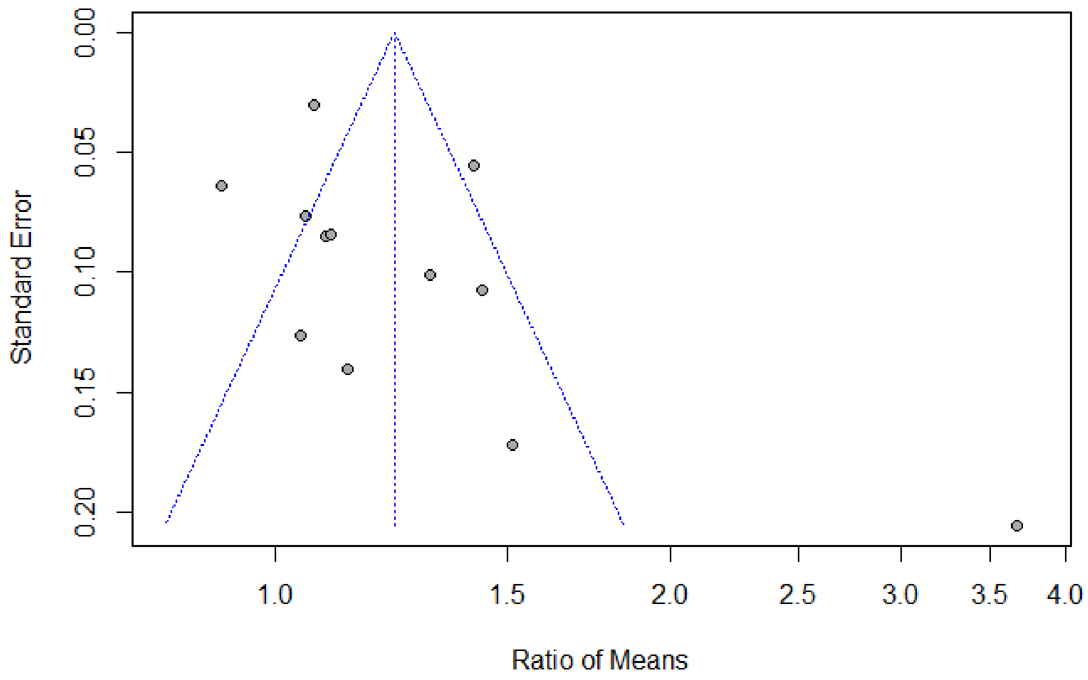

G

Figure S7 Funnel plot of CSF ratios of Ng between Alzheimer's disease (AD) and mild cognitive impairments due to Alzheimer's disease (MCI-AD)

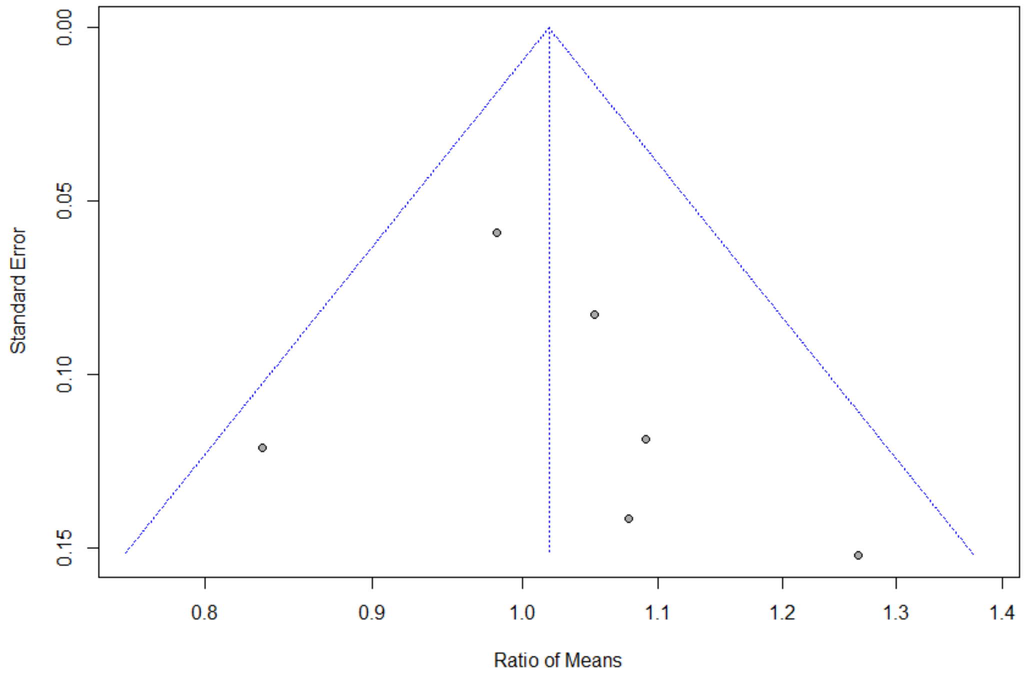

2. Forest plot and funnel plot CSF neurogranin levels using electrochemiluminescence (ECL) in patients with AD and CTRL

Figure S8 CSF ratios of Electrochemiluminescence

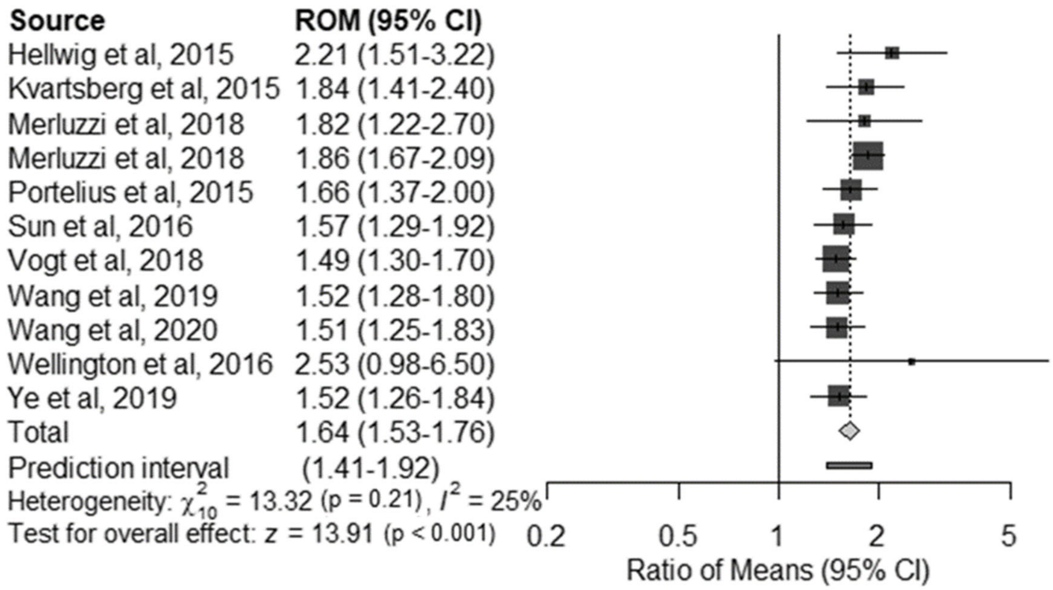

Figure S9 Funnel plot of CSF ratios of Electrochemiluminescence

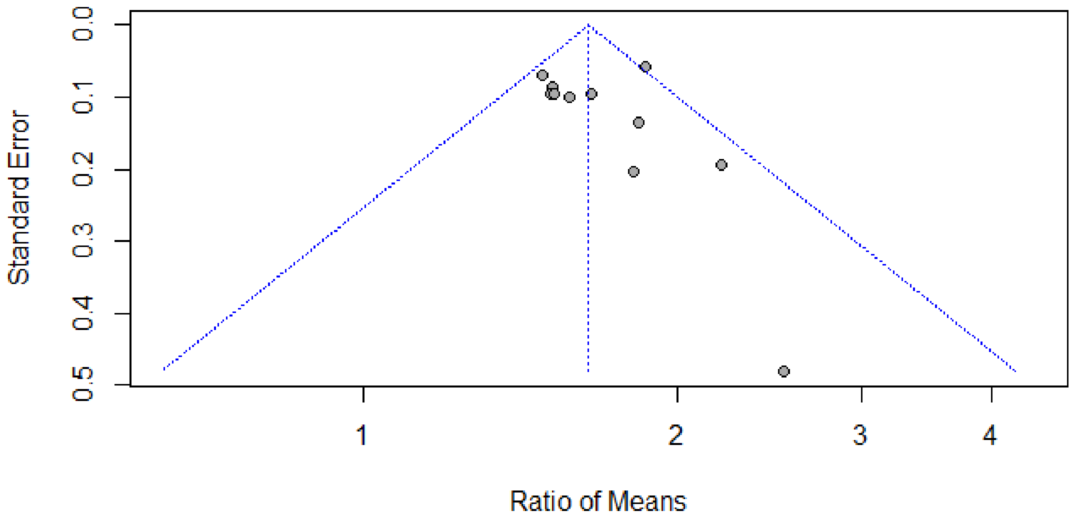

3. Forest plot and funnel plot CSF neurogranin levels using ELISA in patients with AD and CTRL

Figure S10 CSF ratios of ELISA

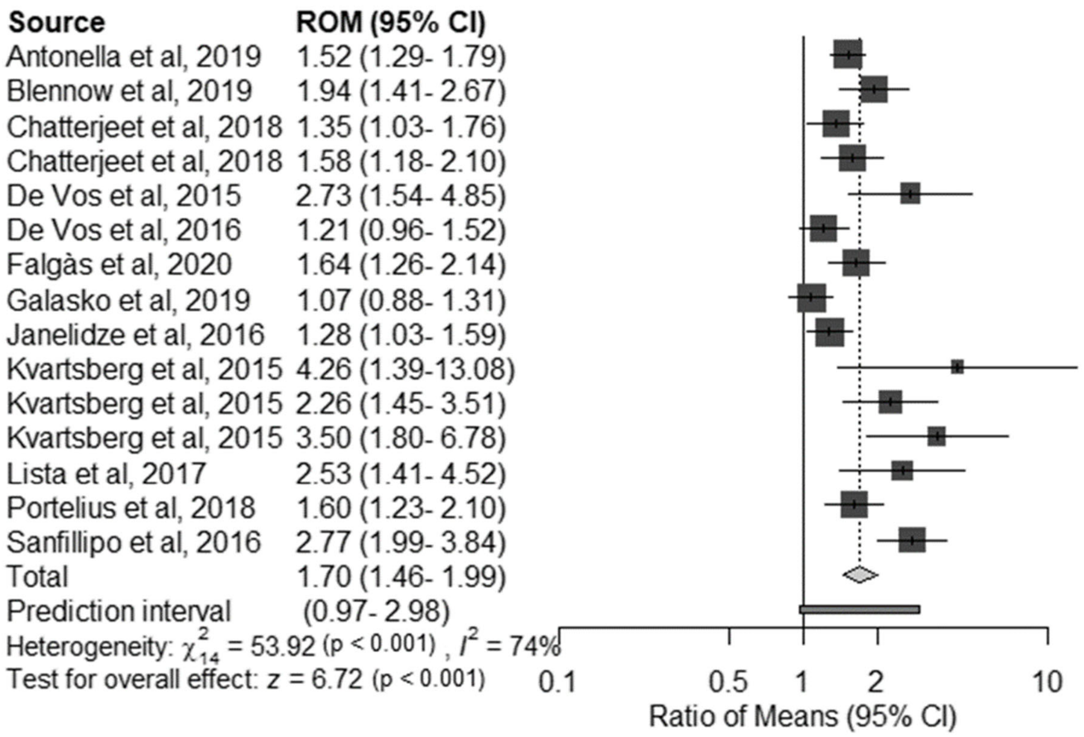

Figure S11 Funnel plot of CSF ratios of ELISA

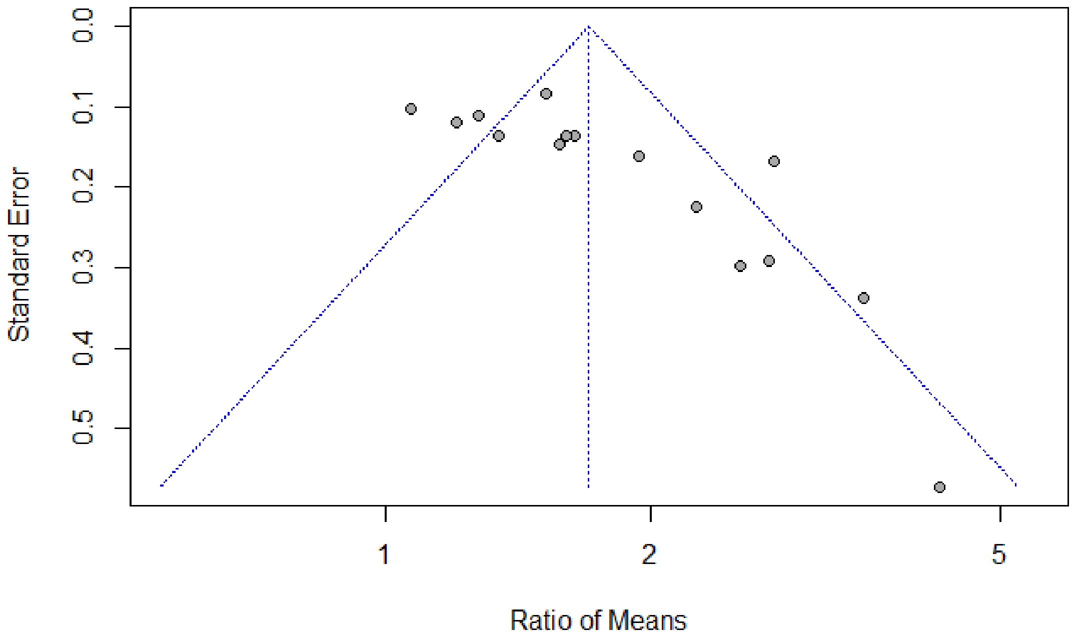

4. Forest plot and funnel plot CSF neurogranin levels using detection antibodies (G62-P75) in patients with AD and CTRL

Figure S12 CSF ratios of Ng (G62-P75)

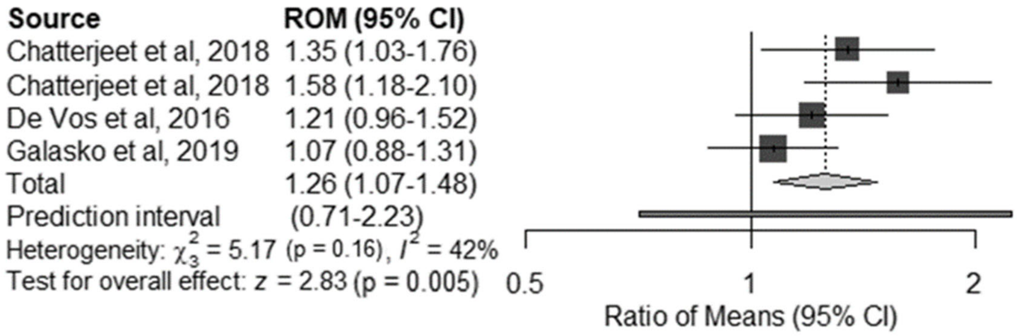

Figure S13 Funnel plot of CSF ratios of Ng (G62-P75)

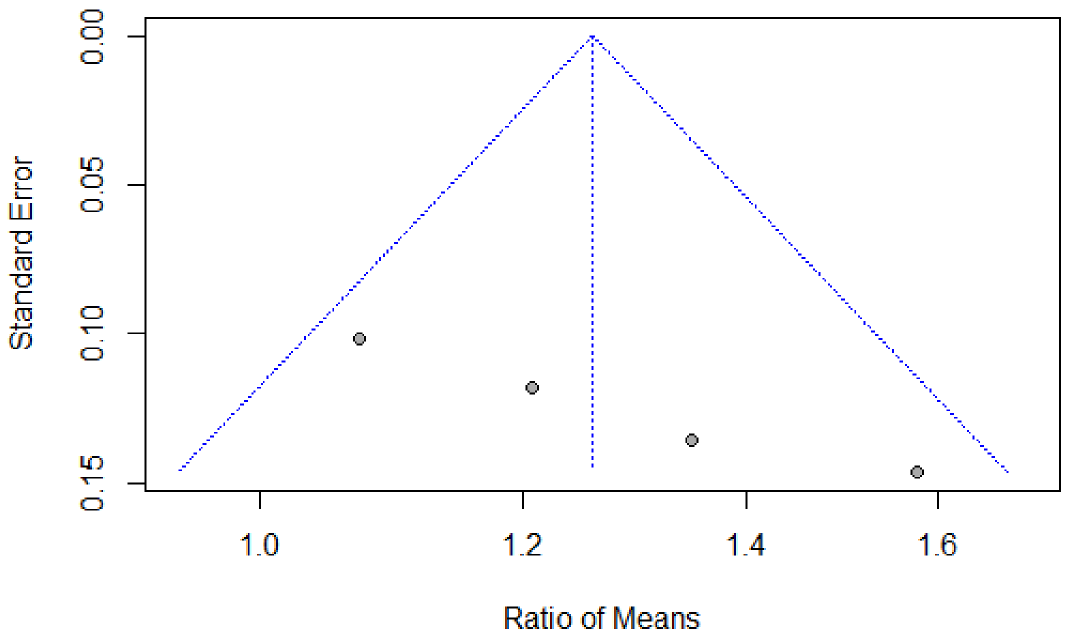

5. Forest plot and funnel plot CSF neurogranin levels using detection antibodies (G62-G65) in patients with AD and CTRL

Figure S14 CSF ratios of Ng7 (G52-G65)

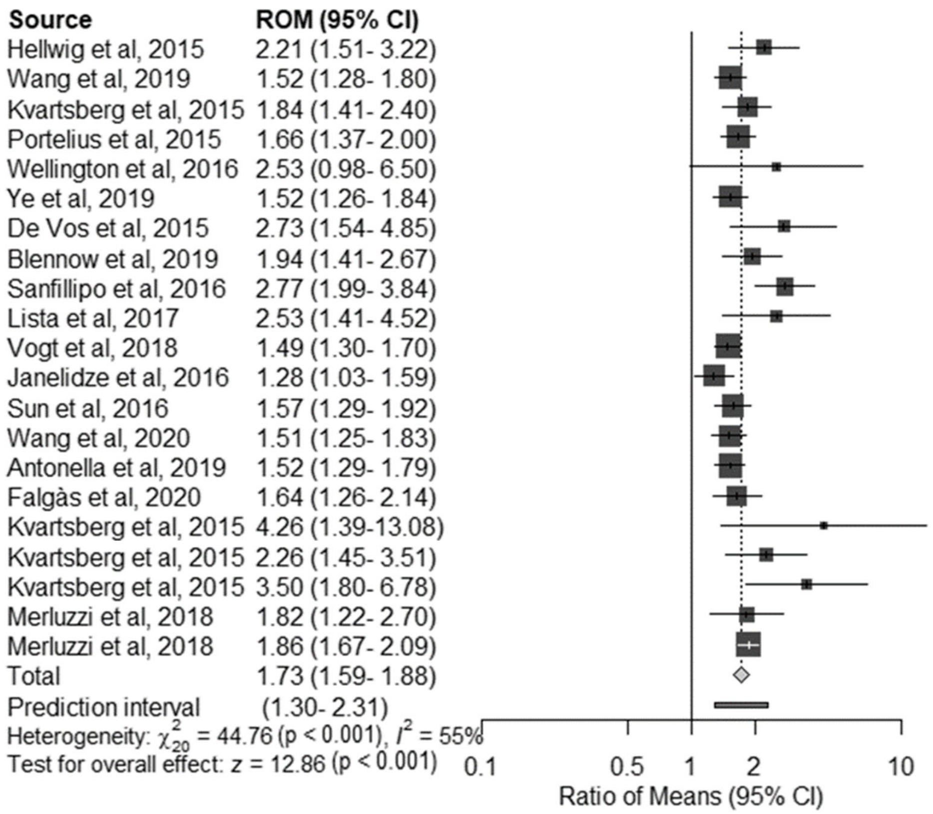

Figure S15 Funnel plot of CSF ratios of Ng7 (G52-G65)

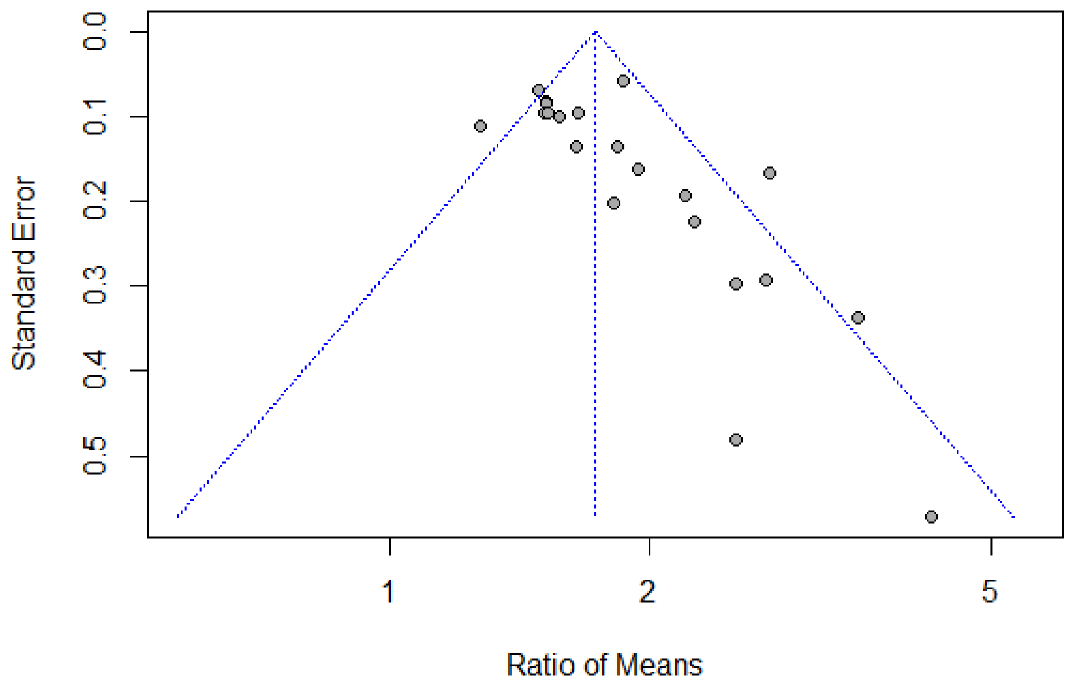

6. Funnel plots of CSF neurogranin levels in patients with compared groups dependent on Aβ status.

A

Figure S16 Funnel plot of CSF ratios of Ng between AD+ group compared to MCI-

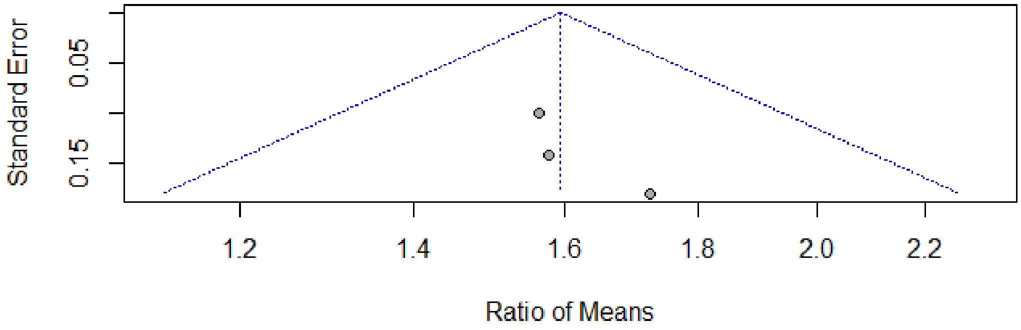

B

Figure S17 Funnel plot of CSF ratios of Ng between AD+ group vs MCI-

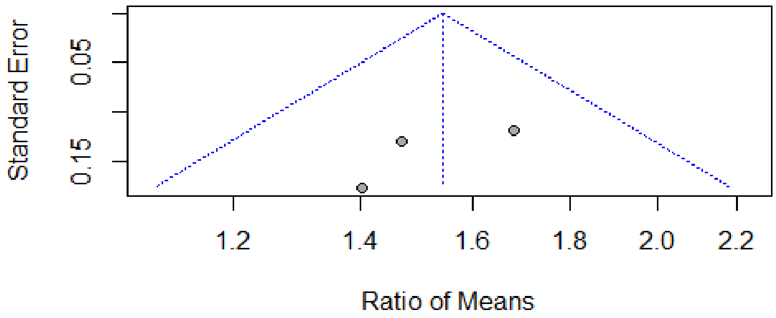

C

Figure S18 Funnel plot of CSF ratios of Ng between MCI+ group vs CTRL-

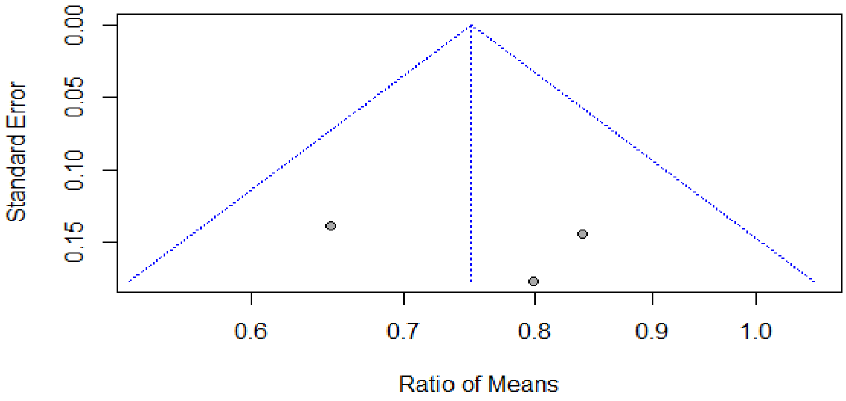

D

Figure S19 Funnel plot of CSF ratios of Ng between MCI+ group compared to CTRL+

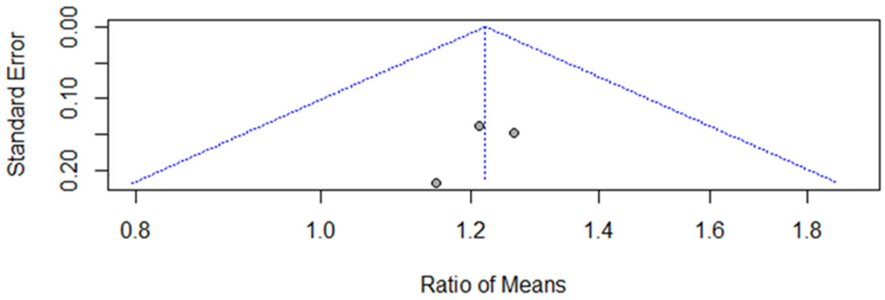

E

Figure S20 Funnel plot of CSF ratios of Ng between AD+ group vs CTRL+

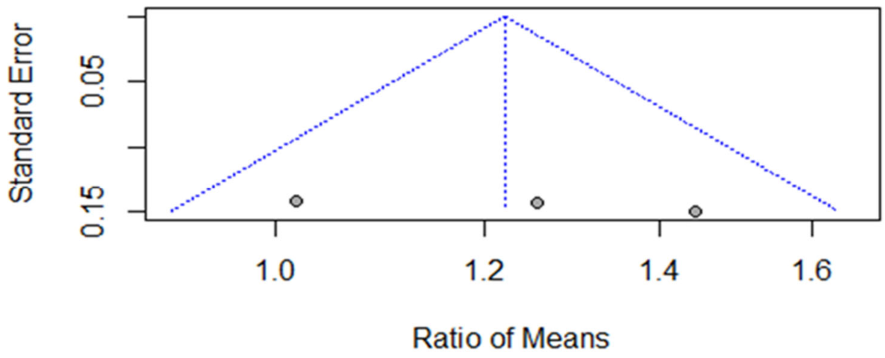

F

Figure S21 Funnel plot of CSF ratios of MCI+ vs CTRL+

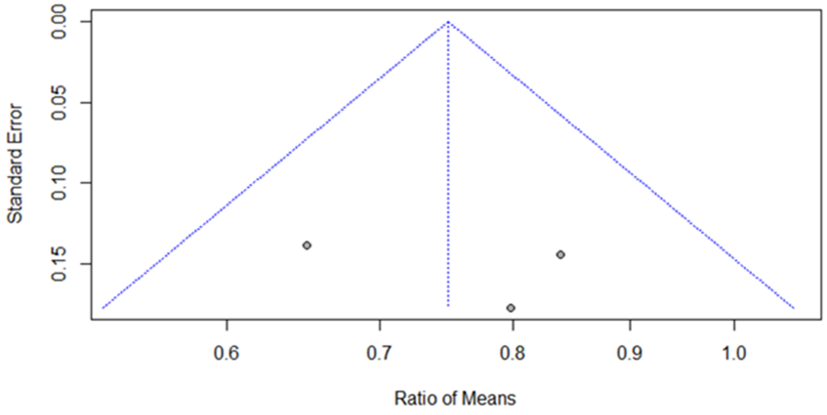

G

Figure S22 Funnel plot of CSF ratios of Ng between AD+ group compared to MCI+

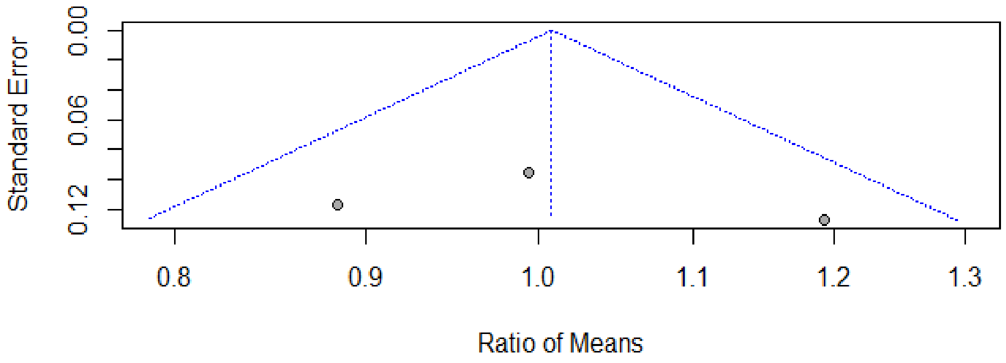

H

Figure S23 Funnel plot of CSF ratios of Ng between MCI- group vs CTRL-

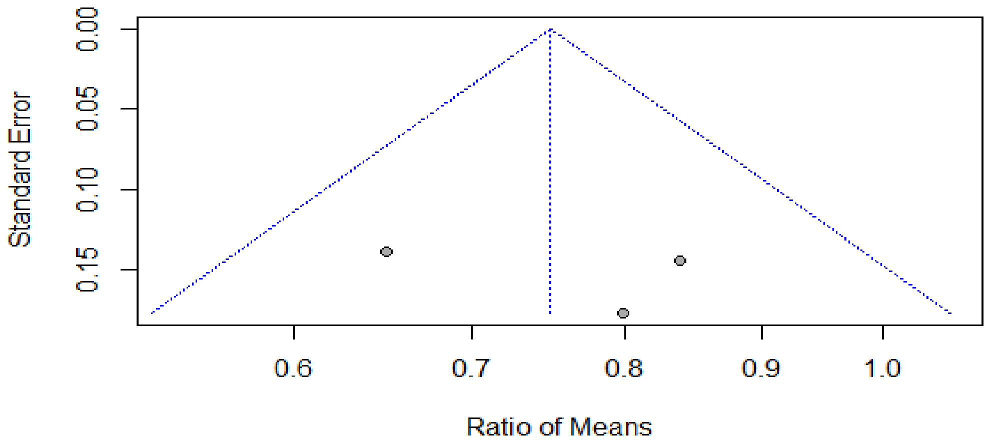

I

Figure S24 Funnel plot of CSF ratios of CTRL+ vs CTRL-

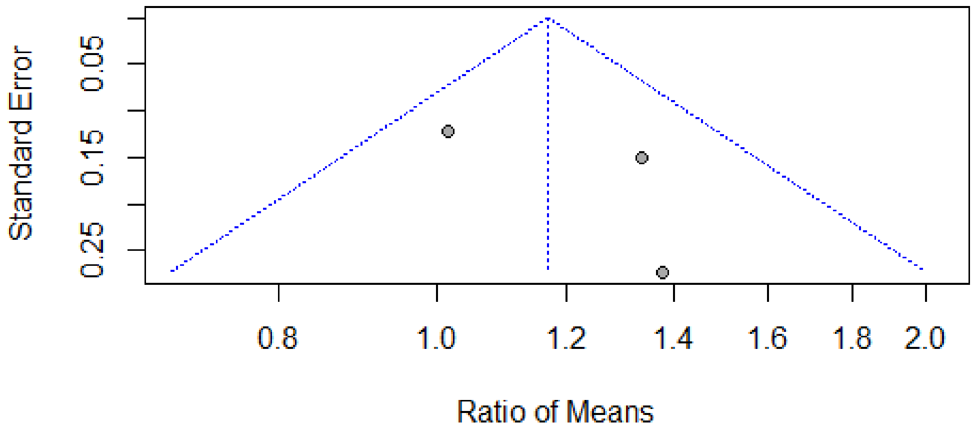

7. Funnel plots with CSF ratios of VILIP-1 for each compared groups.

A

Figure S25 Funnel plot of CSF ratios of VILIP-1 between Alzheimer's disease (AD) and controls (CTRL)

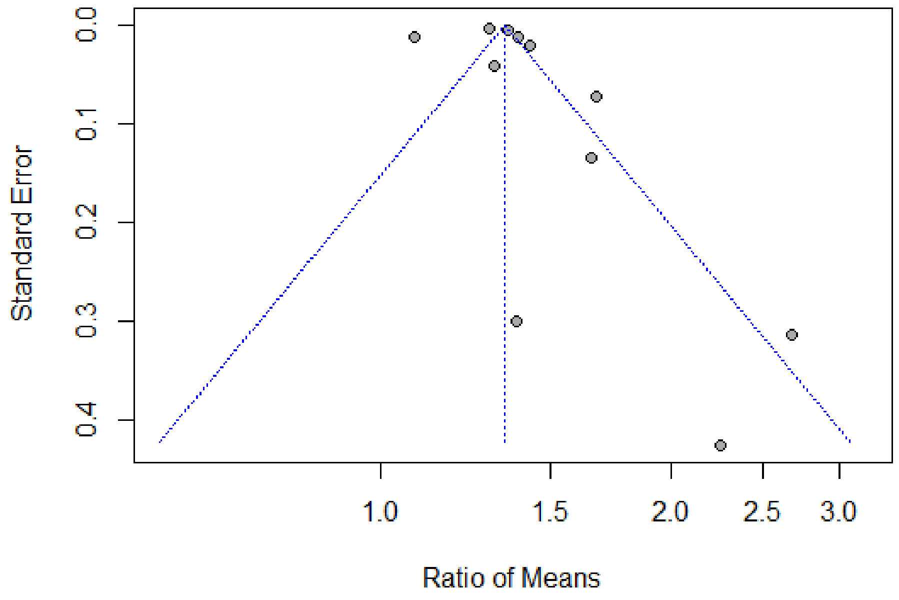

B

Figure S26 Funnel plot of CSF ratios of VILIP-1 between Alzheimer's disease (AD) and mild cognitive impairments (MCI)

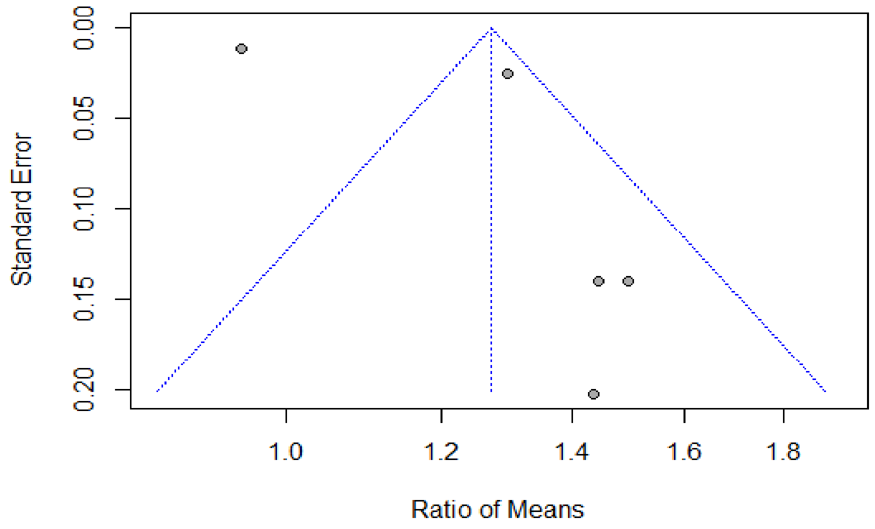

C

Figure S27 Funnel plot of CSF ratios of VILIP-1 between mild cognitive impairments (MCI) and controls (CTRL)

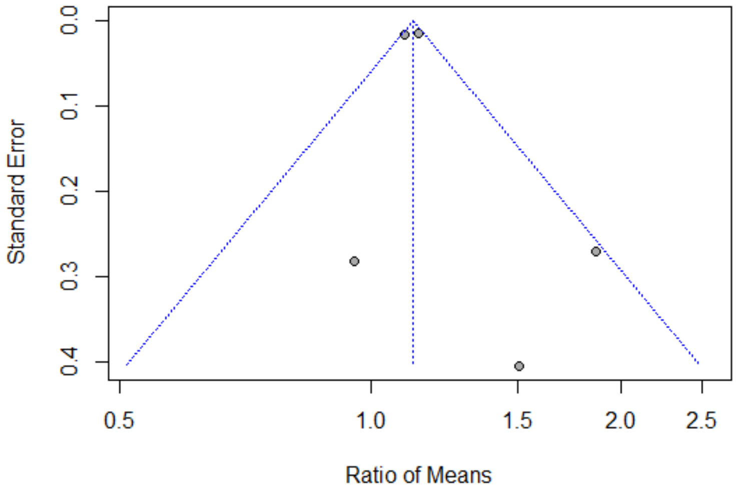

Supplement: Supplementary file 1 [file ijms-21-08335-s001.pdf]
